# Global land projection based on plant functional types with a 1-km resolution under socio-climatic scenarios

**DOI:** 10.1038/s41597-022-01208-6

**Published:** 2022-03-30

**Authors:** Guangzhao Chen, Xia Li, Xiaoping Liu

**Affiliations:** 1 Guangdong Provincial Key Laboratory of Urbanization and Geo-simulation, School of Geography and Planning, Sun Yat-sen University, Guangzhou, China; 2 Institute of Future Cities, The Chinese University of Hong Kong, Shatin, NT Hong Kong SAR; 3 Key Lab of Geographic Information Science (Ministry of Education), School of Geographic Sciences, East China Normal University, Shanghai, China

**Keywords:** Projection and prediction, Climate and Earth system modelling, Environmental impact, Geography

## Abstract

This study presents a global land projection dataset with a 1-km resolution that comprises 20 land types for 2015–2100, adopting the latest IPCC coupling socioeconomic and climate change scenarios, SSP-RCP. This dataset was produced by combining the top-down land demand constraints afforded by the CMIP6 official dataset and a bottom-up spatial simulation executed via cellular automata. Based on the climate data, we further subdivided the simulation products’ land types into 20 plant functional types (PFTs), which well meets the needs of climate models for input data. The results show that our global land simulation yields a satisfactory accuracy (Kappa = 0.864, OA = 0.929 and FoM = 0.102). Furthermore, our dataset well fits the latest climate research based on the SSP-RCP scenarios. Particularly, due to the advantages of fine resolution, latest scenarios and numerous land types, our dataset provides powerful data support for environmental impact assessment and climate research, including but not limited to climate models.

## Abstract (summary)

**Table:** 

Measurement(s)	future land cover
Technology Type(s)	cellular automata • supervised machine learning
Factor Type(s)	spatial driving factors - socioeconomic (GDP, population, urban centre and road) • spatial driving factors - physical (temperature, precipitation, topography and soil quality)

## Background & Summary

Since the industrial revolution, human activities have continuously strengthened in scope and intensity and have substantially impacted regional- and global-scale land-use and land-cover changes (LUCCs)^1,2^. Moreover, this trend is expected continue in the foreseeable future^3,4^. Scenario-based simulations of future land changes can provide important evaluation information on the effect of land policies under different conditions. Thus, they have become a powerful tool for analysing potential future land-use changes^5^. Furthermore, from the ongoing global climate change perspective, scenario-based future land change simulations can provide an essential reference for environmental change risk assessment^6^. Moreover, land simulation products are an essential driving factor for climate models^7^.

To better coordinate international climate research, Phase 6 of the Coupled Model Intercomparison Project (CMIP6) used the latest group of coupled scenarios, the SSP-RCP scenarios^8^. Using these scenarios, different scholars can establish universal and comparable climate studies. In the coupled scenario, shared socioeconomic pathways (SSPs) consider the future social and economic possibilities from population, economy, policy and technology perspectives^9^. Representative concentration pathways (RCPs) employ radiative forcing as an indicator to describe future climate change possibilities^10^. Different SSPs and RCPs can form a scenario matrix comprising numerous coupled scenarios. Therefore, CMIP6 recommends some of the most likely scenarios as critical SSP-RCP scenarios to sharpen the research focus. Herein, we focus on the seven coupling scenarios with the highest priority, Tier 1 and Tier 2 levels, designated by CMIP6, and one coupling scenario that is specially added to achieve the goal of temperature increase below 1.5°C (SSP1-1.9)^11^. Tier 1 level scenarios include SSP1-2.6, SSP2-4.5, SSP3-7.0 and SSP5-8.5, while Tier 2 level scenarios include SSP4-3.4, SSP4-6.0 and SSP5-3.4-OS (hereinafter, abbreviated as SSP5-3.4).

In addition to enriching scenario settings, the resolution of land simulation products needs to be improved. Existing global LUCC prediction products generally suffer from coarse resolution. The resolution of most products is between 5′ and 0.5° (approximately 10–50 km on the equator)^12–14^. For example, for the latest SSP-RCP scenarios, Popp *et al*.^15^ only projected the land area of different regions under SSP without spatial details; the LUH2 dataset^4^ has a coarse resolution of only 0.25°. Chen *et al*.^16^ improved the resolution of the projected land use product to 0.05°, i.e. about 5 km at the equator. Coarse resolutions afford enormous uncertainty to related research using these products, limiting the potential application of these global LUCC projection products^14^. Even at a 10-km resolution, the spatial pattern of urban land will be severely distorted if the resolution is coarse, making it impossible to effectively simulate the spatial changes^17^.

The number of land types included in future land datasets also needs to be improved. Many global and regional climate models (e.g. CLM^7^, ECHAM^18^, RegCM^19^ and WRF^6^) use land cover data with more detailed classification as important driving data^20–22^. Moreover, they require vegetation-type data that can reflect land roughness, surface albedo, soil hydrology and heat characteristics as driving data. Therefore, land data based on plant functional type (PFT) are more suitable for climate research, such as climate models. PFT incorporates numerous land types that can reflect specific ecological functions and climate characteristics^23^. However, most existing future land datasets, especially those with a fine resolution, contain very limited land types. They usually comprise only 6–7 land types; thus, they do not well meet climate models’ requirements^17,24^.

Therefore, this study aims to (1) connect the land projection dataset to the latest group of climate research scenarios, SSP-RCPs, (2) improve the resolution of the global land projection dataset under the latest scenario and (3) enrich future land projection dataset’s land types. Thus, this study first generates a 1-km future global land-use and land-cover (LULC) dataset comprising seven broad land types with 5-year intervals from 2015 to 2100 via land simulation. Then, the LULC dataset is subdivided to afford a PFT-based land dataset containing 20 land types. To our knowledge, this is the highest resolution till date of a future global land dataset with the latest SSP-RCP scenarios. Due to their fine spatial details and rich land type information, the two proposed datasets will contribute to environmental impact assessment and the latest climate research, such as global climate modelling.

## Method

**Fig. 1:**
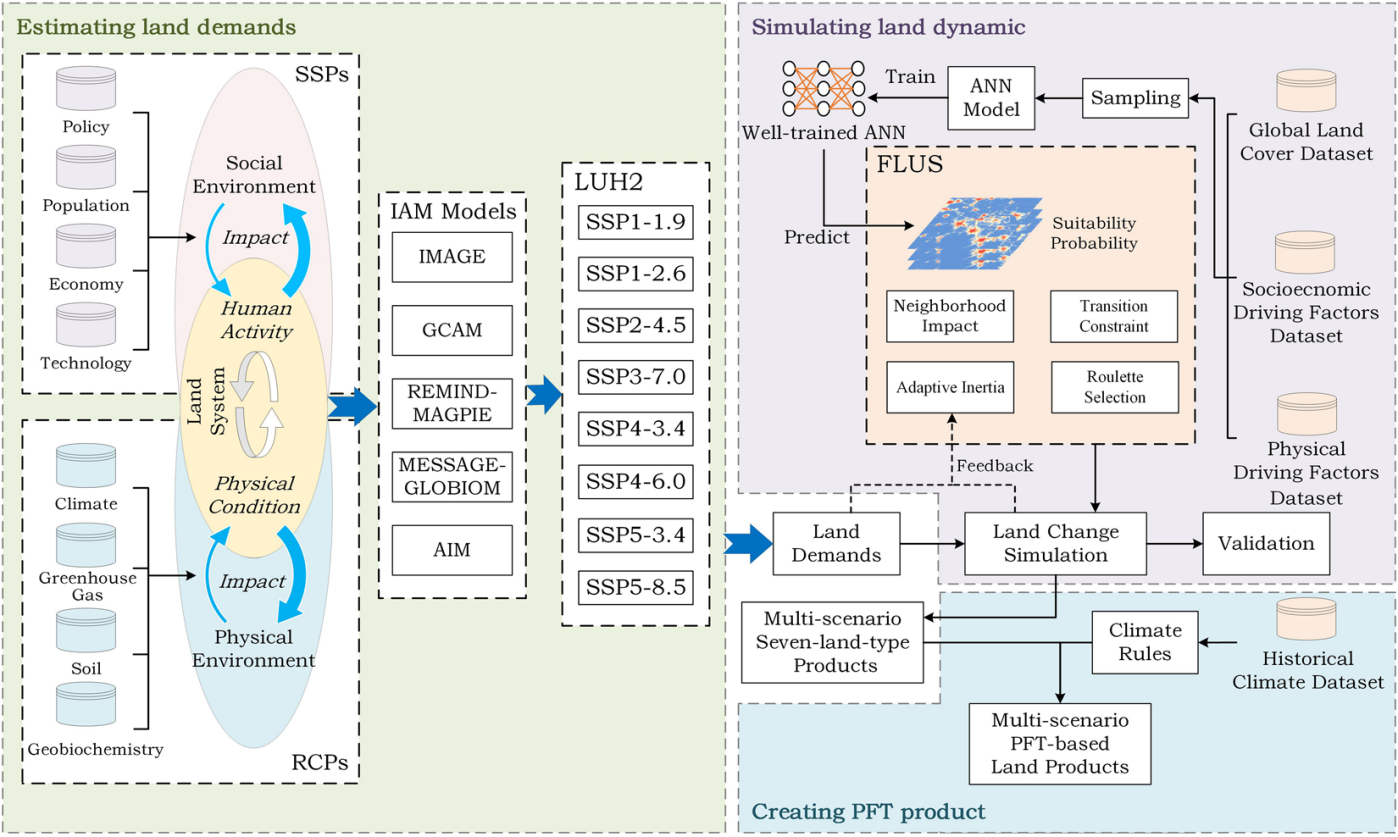
Workflow for creating future global 1-km resolution land datasets of SSP-RCP scenarios.

Fig. 1 shows the creation process of a future land dataset. The process can be divided into three parts. The first part is the estimation of the future area demands for different land types under different SSP-RCP scenarios. The second part is the implementation of a 1-km spatial land simulation using the future land use simulation (FLUS) model under the macro constraints of the demands. The FLUS model is discussed below. At this point, a future LULC dataset containing seven land types is afforded. Furthermore, in the third part, we subdivide the land types to form a future land dataset of SSP-RCP scenarios with a 1-km resolution based on PFT classification.

### Estimating future land demand

CMIP6 affords a set of officially recommended future land-use datasets, LUH2 (Land-Use Harmonization 2)^4^, which is available for free at http://luh.umd.edu/index.shtml. We downloaded the LUH2 v2f Release with tree cover correction files on 25 February 2019. This dataset comprises a global projection of multiple land types for successive years from 2015 to 2100 under different SSP-RCP scenarios with a 0.25° resolution (approximately 25 km at the equator). Considering CMIP6, the recommended land-use prediction results for different SSP-RCP scenarios have been provided using different integrated assessment models (IAMs) created by different research teams^25^. IAMs are a class of integrated models that integrate relevant models developed in various disciplines, such as energy, economics, atmospheric chemistry, climate and ecology by constructing representative sectors^26^. One IAM can project several SSP-RCP scenarios^3,27–30^.

In the official future land-use datasets, however, a specific SSP only corresponds to a recommended IAM, which also affords RCPs coupling with this SSP, i.e. IMAGE model for SSP1^31^, MESSAGE-GLOBIOM model for SSP2^29^, AIM model for SSP3^28^, GCAM model for SSP4^30^ and REMIND-MAGPIE model for SSP5^27^. Notably, LUH2 integrates the results afforded by different IAMs so that they have the same initial land-use distribution. Nevertheless, specific conversions are required before it can be used to support our land simulation because of its particular land classification and coarse resolution.

First, we mapped the land classification from LUH2 to the classification used in our simulation, which is based on the ESA-CCI land cover data from 2015. Table 1 shows the specific corresponding relation. The land types in LUH2 are combined into seven classes, wherein *water* and *permanent ice and snow* remain unchanged in the simulation.

**Table 1:** Land classification relations among LUH2, ESA-CCI and ours.

Land classification in our simulation	Land classification in LUH2	Land classification in ESA-CCI
Forest	Forested primary land	Tree cover, broadleaved, evergreen
	Potentially forested secondary land	Tree cover, broadleaved, deciduous
		Tree cover, needleleaved, evergreen
		Tree cover, needleleaved, deciduous
		Tree cover, mixed leaf type
		Shrubland
		Mosaic tree and shrub (>50%)/herbaceous cover (<50%)
		Mosaic natural vegetation (tree, shrub, herbaceous cover) (>50%)/cropland (<50%)
Grassland	Managed pasture	Grassland
	Rangeland	Mosaic herbaceous cover (>50%)/tree and shrub (<50%)
Barren	Non-forested primary land	Bare areas
	Potentially non-forested secondary land	Lichens and mosses
		Sparse vegetation (tree, shrub, herbaceous cover) (<15%)
Cropland	C3 annual crops	Cropland, rainfed
	C3 perennial crops	Cropland irrigated or post-flooding
	C4 annual crops	Mosaic cropland (>50%)/natural vegetation (tree, shrub, herbaceous cover) (<50%)
	C4 perennial crops	
	C3 nitrogen-fixing crops	
Urban	Urban land	Urban areas
Water	—	Water bodies
Permanent snow and ice	—	Permanent snow and ice

Second, statistics on the land demand trends in LUH2 for each scenario and region were compiled. We divided the world into 31 regions by referring to the SSP official database’s partitions^32^ (https://tntcat.iiasa.ac.at/SspDb, accessed on 20 September 2018, see Figure S1). The partition principles were mainly as follows: First, since countries with different development states tend to adopt different policies, countries with similar development status were divided into the same region. Second, considering spatial heterogeneity, spatially neighbouring countries were preferentially divided into the same region. To deal with the area gap between LUH2 and ESA-CCI land cover data in the initial year, 2015, the land change trends from LUH2 were extracted to calibrate the future land demands.

The calibration can be further subdivided: preliminary calibration and harmonisation of the total area. The preliminary future land demand based on the 2015 ESA-CCI land cover data was calibrated considering the land change trend in LUH2. The equation is as follows:

1 $$\left\{\begin{array}{lll}Are{a{\prime} }_{r,j}^{t} & = & Are{a{\prime} }_{r,j}^{t-1}\times \left(\Delta rat{e}_{r,j}^{t}\times \frac{LUH{2}_{r,j}^{t-1}}{Are{a{\prime} }_{r,j}^{t-1}}+1\right),\quad \frac{LUH{2}_{r,j}^{t-1}}{Are{a{\prime} }_{r,j}^{t-1}} < 1\;and\;j\ne urban\\ Are{a{\prime} }_{r,j}^{t} & = & Are{a{\prime} }_{r,j}^{t-1}\times \Delta rat{e}_{r,j}^{t},\quad \frac{LUH{2}_{r,j}^{t-1}}{Are{a{\prime} }_{r,j}^{t-1}}\ge 1\;and\;j\ne urban\end{array}\right.$$

2 $$\left\{\begin{array}{lll}Are{a{\prime} }_{r,urban}^{t} & = & Are{a{\prime} }_{r,urban}^{t-1}\times \Delta rat{e}_{r,urban}^{t},\quad \Delta rat{e}_{r,urban}^{t}\ge 1\\ Are{a{\prime} }_{r,urban}^{t} & = & Are{a{\prime} }_{r,urban}^{t-1},\quad \Delta rat{e}_{r,urban}^{t} < 1\end{array}\right.$$

where $Are{a{\prime} }_{r,j}^{t}$ represents the preliminary calibrated demand for land type ***j*** in region ***r*** at time ***t***. Additionally, $\Delta rat{e}_{r,j}^{t}$ denotes the net change rate of the area of land type ***j*** in region ***r*** from time ***t−1*** to ***t*** in LUH2. $LUH{2}_{r,j}^{t-1}$ denotes the area of land type ***j*** in region ***r*** at time ***t−1*** in LUH2. Through such calibration, the illogical drastic fluctuations of land demands caused by the difference in the initial area in different products can be reduced while maintaining the original trend of LUH2. As an exception, Eq. 2 was applied to urban land since it represents a small fraction of the land, and this study assumes that urban land does not shrink at the 1-km scale.

Second is the harmonisation of the total land area. The total land area after preliminary calibration may be inconsistent with the actual total land area. Therefore, we adjusted the areas of the various land types obtained from the preliminary calibration using a proportion-based approach to render their total areas equal to the actual total land areas. Note that urban land was not considered in the adjustment because of its small proportion. The equation for the adjustment is as follows:

3 $$\left\{\begin{array}{lll}Are{a}_{r,j}^{t} & = & \left(Are{a}_{r}^{total}-Are{a}_{r,U}^{t}\right)\times \frac{Are{a{\prime} }_{r,j}^{t}}{{\sum }_{J}Are{a{\prime} }_{r,j}^{t}},\quad j\ne urban\\ Are{a}_{r,j}^{t} & = & Are{a{\prime} }_{r,j}^{t},\quad j=urban\end{array}\right.$$

where $Are{a}_{r,j}^{t}$ represents the adjusted demand for land type ***j*** in region ***r*** at time ***t***, *U* denotes urban land and $Are{a}_{r}^{total}$ represents the actual total land area of region ***r***. The adjusted future land-use demands were used as simulation targets for different scenarios and as constraint conditions for iteration termination when performing future land-use simulations.

### Simulating future land dynamic

Herein, we used the FLUS model to simulate future land-use dynamics. It is a widely used land simulation model that effectively simulates land-use change at global and regional scale^33–37^. Furthermore, it can be coupled with IAMs and system dynamics models. It has been successfully applied to the long-term simulation of global land cover change under the SRES scenario^17^ and the simulation of global urban land change under SSPs from 2015 to 2100^38^, reflecting its reliable computing capabilities. Compared to traditional cellular automata (CA), the FLUS model has the following advantages: First, it uses a roulette selection mechanism to determine the state of each cell changes, which can adequately reflect the competition and randomness of various land types in reality. Simultaneously, it eliminates the drawbacks of traditional CA, which requires researchers to subjectively set thresholds. Second, the FLUS model adopts adaptive inertia coefficients; thus, the iteration speed can be automatically adjusted according to the difference between the existing land area and the target land area after each iteration. Thereby, the FLUS model eliminates the subjective setting of iterative speed parameters in traditional CA. Equation 4 expresses the execution of the FLUS model^37^:

4 $$T{P}_{i,j}=P{g}_{i,j}\times neighbo{r}_{i,j}\times inerti{a}_{j}\times con{s}_{k\to j}$$

where $T{P}_{i,j}$ represents the total probability of grid cell ***i*** becoming land type ***j***. $P{g}_{i,j}$ represents the suitability probability of land type ***j*** on grid cell ***i***. $neighbo{r}_{i,j}$ represents the neighbourhood effect of land type ***j*** around grid cell ***i***, and it is positively related to the number of grids of land type ***j*** around grid cell ***i***. Moreover, $inerti{a}_{j}$ represents the adaptive inertia coefficient of land type ***j***, and $con{s}_{k\to j}$ represents the constraint of changing from the current land type ***k*** to land type ***j***. That is, its value is 1 when such conversion is allowed; otherwise, it is 0. Herein, water bodies and permanent ice and snow are frozen, and urban land cannot change to other land types.

The estimation of the suitability probability is the key for ensuring the effective execution of the FLUS model. The FLUS model employs artificial neural networks (ANNs) to train and estimate the suitability probabilities of various land types^37^. Since our research comprises numerous land types, including artificial land (such as urban) and natural land (such as woodland, grassland and wasteland), we need to select appropriate spatial driving factors and input them into the ANNs to drive the suitability probability estimation. Regarding the existing land simulation studies^5,39–41^, we selected a series of relevant spatial driving factors, such as socioeconomic (GDP, population, urban centre and road) and physical (temperature, precipitation, topography and soil quality) factors. We selected the driving factors that are close in time to the initial year of our simulation. Furthermore, the driving factors’ original resolutions are as close as possible to 1 km, except for the soil quality factor (5′ resolution). Nevertheless, since the soil’s spatial heterogeneity is not as prominent as factors such as population and GDP, a 5′ resolution (approximately 10 km on the equator) is also acceptable. Table 2 shows the spatial driving factors used herein. All these factors were resampled to 1-km resolution before being input to the ANN for training and evaluation.

**Table 2:** Spatial driving factors used in this study.

Spatial Variables	Year	Resolution	Data Sources
GDP	2006	1 km	Ghosh *et al*.^50^
Population	2010	0.5′	LandScan 2010 Global Population Project^51^
Human Influence Index	2004	0.5′	NASA Socioeconomic Data and Applications Center, Global Human Influence Index, v2^52^
Distance to cities (population > 30 × 10^3^)	2014	1 km	United Nations, Department of Economic and Social Affairs, Population Division (2014)^53^
Distance to roads	1980-2010	1 km	NASA, Socioeconomic Data and Applications Center, Global Roads Open Access Data Set (gROADS), v1^54^
DEM	2000	0.5′	Hijmans *et al*.^55^
Slope	2000	0.5′	Calculated from DEM by Slope tool provided by ArcGIS software
Annual Mean Temperature	2000	0.5′	Hijmans *et al*.^55^
Temperature Annual Range			
Temperature Seasonality			
Annual Precipitation	2000	0.5′	Hijmans *et al*.^55^
Precipitation Seasonality			
Soil quality (Excess salts)	2008	5′	Fischer *et al*.^56^
Soil quality (Nutrient availability)			
Soil quality (Oxygen availability to roots)			
Soil quality (Workability)			

The FLUS model software (GeoSOS-FLUS V2.4)^37^ for performing future land change simulations can be downloaded for free from http://www.geosimulation.cn/FLUS.html. The FLUS Model module of the software can implement the operations of this subsection. First, we estimated each land type’s suitability probability (also called *Probability-of-occurrence*) in each region separately by inputting the corresponding land data and spatial driving factors. Then, we executed the land use simulation by region using the FLUS model under the SSP-RCPs land demand constraints. The simulation was performed with a 1-km resolution for 2015–2100, with 5-year intervals.

### Creating plant functional types (PFT) product

**Fig. 2:**
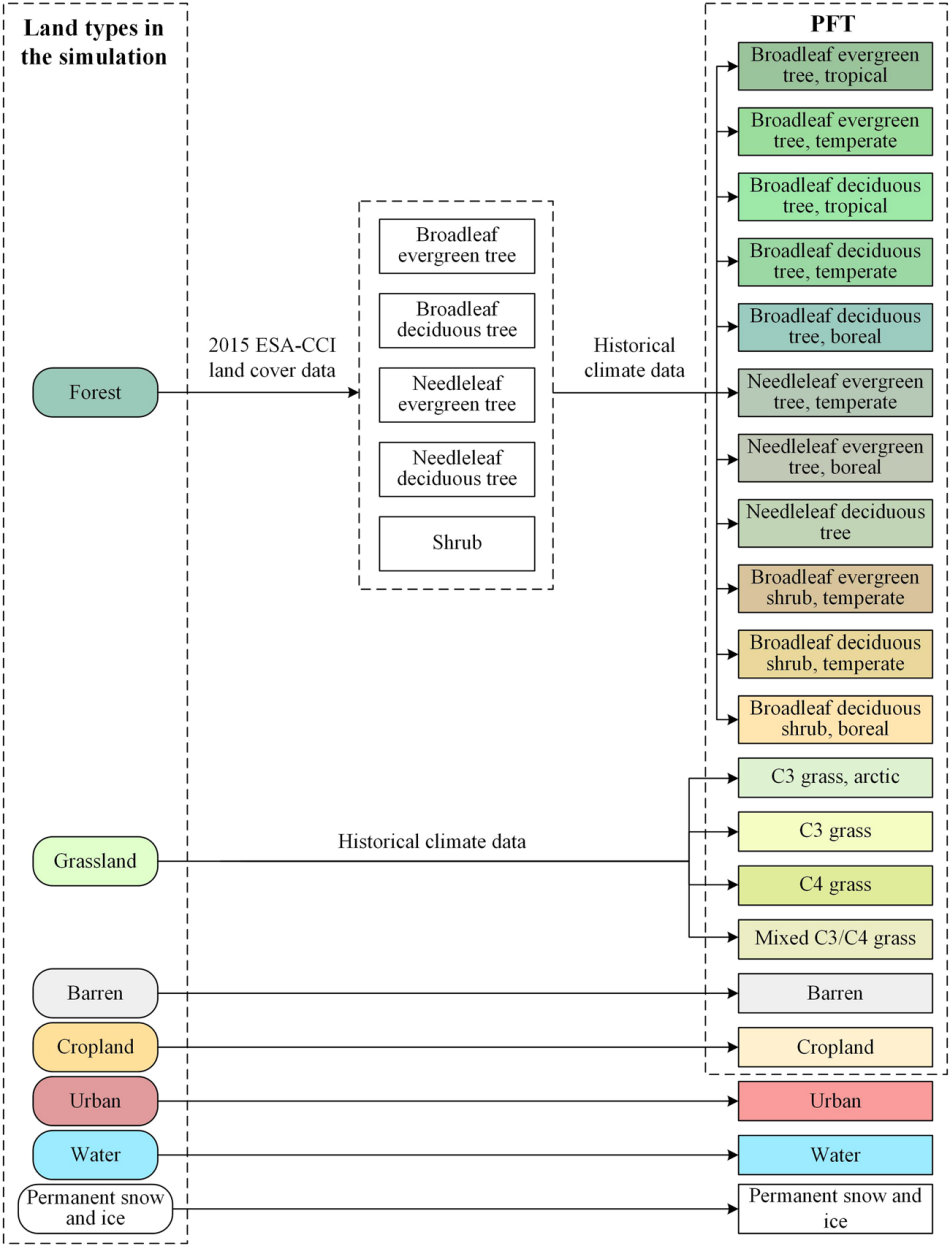
Workflow for the 1-km future global land cover products based on PFT classification.

The PFT classification used in the CLM model^7^ is used as a reference to create our future global PFT dataset based on previous land simulation results. Each grid unit comprises five landunits in the CLM model: glacier, lake, wetland, vegetated and urban. Since we do not simulate the changes of glaciers and water bodies, the first three landunits can be frozen in the initial year data of the ESA-CCI dataset. Next, the CLM model subdivides the vegetated types, especially forest and grassland, into 15 PFTs. Thus, we referred to the method proposed by Bonan *et al*.^42^ and used historical average climate data and coarse-classified vegetated distribution data for subdividing forest and grassland into 15 corresponding PFTs. Subsequently, a future global land cover product based on PFT classification containing 20 land types was afforded. Figure 2 shows the workflow.

As shown in Figure 2, barren, cropland, urban, water and permanent snow and ice can be directly retained from the previous land simulation results. Therefore, the land subdivision based on PFT can be divided into two parts: forests and grasslands.

#### Subdivision for forest-type PFTs

Forest-type PFTs were subdivided into two steps. First, based on the 2015 ESA-CCI land cover data and the nearest neighbour principle, each forest-type grid in the future years in each SSP-RCP scenario was assigned to one of the five preliminary forest-type PFTs (i.e. broadleaf evergreen tree, broadleaf deciduous tree, needleleaf evergreen tree, needleleaf deciduous tree and shrub). The nearest neighbour principle is the most straightforward method for judging the potential of forest-type PFT. Second, the five preliminary PFTs were further subdivided into 11 types of forest-type PFTs using historical average climate data. The 1-km global historical average climate dataset provided by WorldClim^43^ (version 2.0, download on 3 July 2018, https://worldclim.org/) was employed. This dataset comprises the average monthly climate indicators for 30 years (1970–2000), such as average temperature, maximum temperature, minimum temperature, precipitation and solar radiation. Additionally, to achieve reliable accuracies, it uses climate information from 9,000 to 60,000 weather stations worldwide and the MODIS platform^43^.

**Fig. 3:**
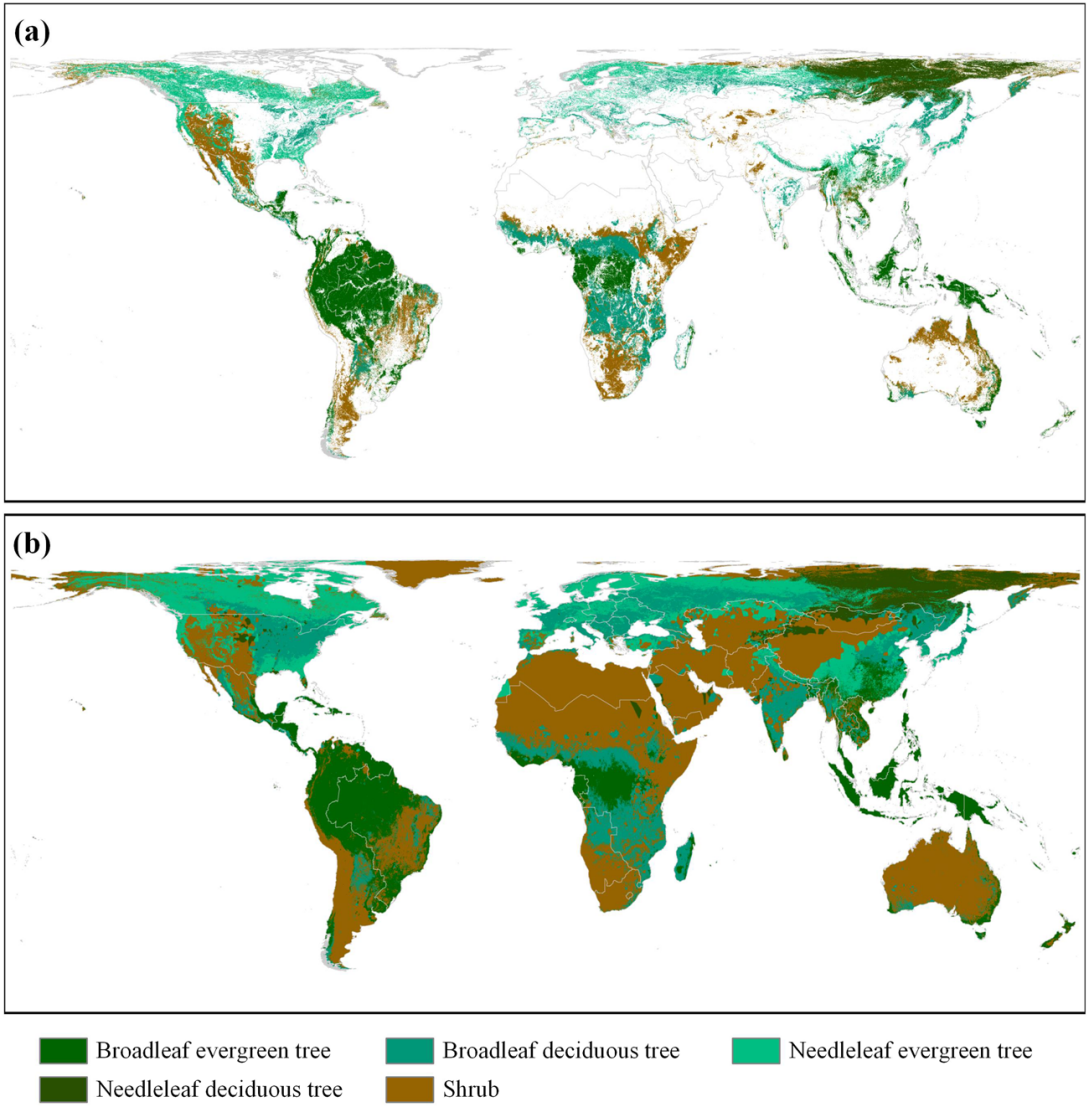
The basis and results of the preliminary estimation of the potential distribution of forest-type PFTs. (**a**) The distribution of forest-type PFTs in ESA-CCI in 2015. (**b**) The preliminary potential distribution of forest-type PFTs based on (**a**) and the nearest neighbour principle.

The effect of the first step is shown in Fig. 3. After the preliminary forest-type PFTs were subdivided, the method proposed by Bonan *et al*.^42^ was used to further subdivide them into 11 forest-type PFTs according to different climate rules (Table 3).

**Table 3:** Subdivision rules for forest-type PFTs.

Preliminary forest-type PFT	Subdivided forest-type PFT	Climate rules
Broadleaf evergreen tree	Broadleaf evergreen tree, tropical	T_c_ > 15.5°C
Broadleaf evergreen tree	Broadleaf evergreen tree, temperate	T_c_ ≤ 15.5°C
Broadleaf deciduous tree	Broadleaf deciduous tree, tropical	T_c_ > 15.5°C
Broadleaf deciduous tree	Broadleaf deciduous tree, temperate	−15°C<T_c_ ≤ 15.5°C and GDD > 1200
Broadleaf deciduous tree	Broadleaf deciduous tree, boreal	T_c_ ≤ −15°C or GDD ≤ 600
Needleleaf evergreen tree	Needleleaf evergreen tree, temperate	T_c_ > −19°C and GDD > 600
Needleleaf evergreen tree	Needleleaf evergreen tree, boreal	T_c_ ≤ −19°C or GDD ≤ 600
Needleleaf deciduous tree	Needleleaf deciduous tree	None
Shrub	Broadleaf evergreen shrub, temperate	T_c_ > −19°C and GDD > 600 and P_ann_ > 520 mm and P_win_ > 2/3 P_ann_
Shrub	Broadleaf deciduous shrub, temperate	T_c_ > −19°C and GDD > 600 and (P_ann_ ≤ 520 mm or P_win_ ≤ 2/3 P_ann_)
Shrub	Broadleaf deciduous shrub, boreal	T_c_ ≤ −19°C or GDD ≤ 600

In Table 3, T_c_ is the average temperature in the coldest month, T_w_ is the average temperature in the warmest month, P_ann_ is the average annual precipitation, P_win_ is the average precipitation in the winter half year (referring to November–April of the following year in the northern hemisphere and May–October in the southern hemisphere) and GDD (growing-degree day) is the annual accumulated temperature of days over 5°C^42^. The daily GDD, $GD{D}_{d}$, can be expressed as follow:

5 $$GD{D}_{d}=\left\{\begin{array}{cc}{T}_{d}-{T}_{b}, & {T}_{d} > {T}_{b}\\ 0, & {T}_{d}\le {T}_{b}\end{array}\right.$$

GDD is the sum of $GD{D}_{d}$ in one year. ${T}_{d}$ is the daily average temperature. ${T}_{b}$ is the base temperature of crop development, which is set as 5°C here. However, the WorldClim dataset does not provide historical daily average temperatures. Therefore, when calculating GDD, a trade-off method was adopted. That is, ${T}_{d}$ was replaced with the monthly average temperature, and then, the calculated $GD{D}_{d}$ was multiplied by the number of days in the corresponding month. The historical average climate data used for subdividing forest-type PFTs are shown in Figure S2~S6.

#### Subdivision for grassland-type PFTs

Specific climatic rules were also adopted to subdivide grassland-type PFTs. Grassland-type PFTs were subdivided into arctic C3 grass, C3 grass and C4 grass in the CLM model. Different grasslands are located in regions with different climatic characteristics. Considering the method proposed by Bonan *et al*.^42^, we used the climate rules shown in Table 4 to subdivide the grassland-type PFTs.

**Table 4:** Subdivision rules for grassland-type PFT.

Preliminary grassland-type PFT	Subdivided grassland-type PFT	Climate rules
Grassland	C3 grass, arctic	GDD<400
Grassland	C3 grass	GDD ≥ 400 and (T_w_ ≤ 22°C or six months P_mon_ ≤ 25 mm and for month T_mon_ > 22°C)
Grassland	C4 grass	GDD ≥ 400 and T_c_ ≥ 22°C and driest month P_mon_ > 25 mm
Grassland	Mixed C3/C4 grass	Other grasslands that do not meet the above rules

In Table 4, P_mon_ represents monthly precipitation. Additionally, the meanings and corresponding data of other abbreviations are the same as in Table 3. Mixed C3/C4 grass denotes that both C3 and C4 grasses account for 50% of a 1-km grid. The historical average data of precipitation in the driest month are shown in Figure S7.

## Data Records

Two land datasets from 2015 to 2100 with 5-year intervals were created herein under the following SSP-RCP scenarios: 1) 1-km global land dataset comprising seven land types and 2) 1-km global PFT-based land dataset comprising 20 land types. Both land datasets are publicly available and open source at 10.5281/zenodo.4584775^44^ or http://www.geosimulation.cn/Global-SSP-RCP-LUCC-Product.html. All files in the datasets are in single-band GeoTIFF format, representing one year of a scenario. GeoTIFF files can be processed in ArcGIS and using programming languages such as Python. Moreover, some extension packages, such as the GDAL package for Python, can make the handling of these GeoTIFF files easy.

## Technical Validation

### Future land demand

**Fig. 4:**
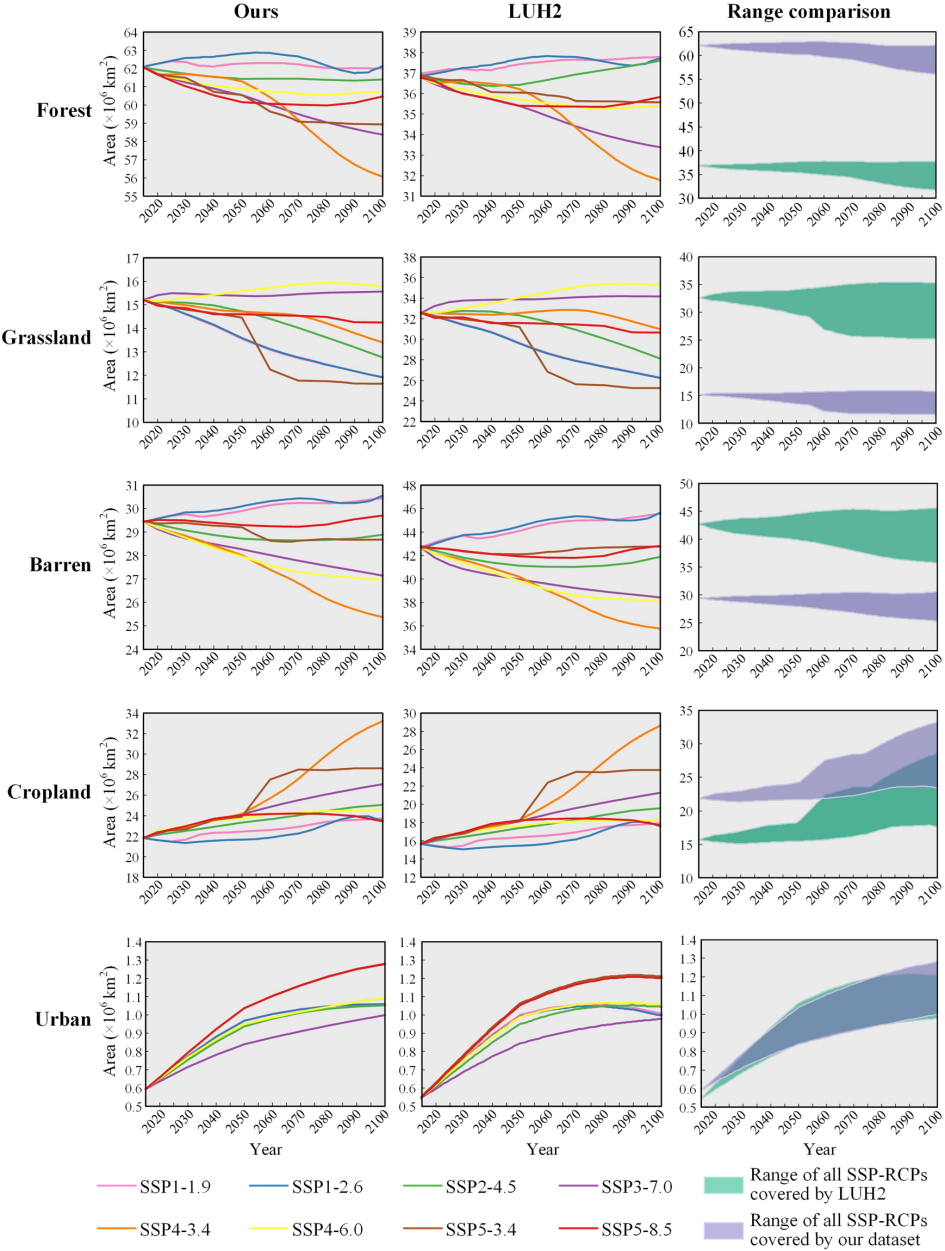
Comparison of demands for major land types between ours and LUH2 under the SSP-RCP scenarios on a global scale (2015–2100).

After extracting and calibrating the LUH2 dataset, we obtained the demands for each land type in the 31 regions of the world from 2015 to 2100 under the SSP-RCP scenarios used during the land change simulations. To demonstrate the effect of calibrating the land demands provided by LUH2, the land demands and their trajectories were compared on a global scale (Fig. 4).

As shown in Fig. 4, the two-step calibration of LUH2 data yields satisfactory results. The results show that our adjusted land demands for each land type in each scenario are maintained highly similar to that in LUH2. That is, the trajectory of each land type in each scenario in LUH2 is well preserved. Moreover, for the gaps between our dataset and LUH2 in the area of different land types in the initial year, with some relatively large gaps, the change ranges of our adjusted land demands are comparable to LUH2. Therefore, the land demands in our land change simulation fully reflect the macro constraints on land change caused by SSP-RCPs’ storyline. Simultaneously, these projected demands are consistent and comparable with land projections made by various IAMs in CMIP6.

### Validation of the land simulation

To validate the land simulation accuracy, historical simulations were independently conducted on the 31 regions and the obtained accuracies were evaluated. The historical simulation spanning from 1992 to 2015 reflects the long-term simulation performance of our model. The land data observed in 1992 also stem from the ESA-CCI land dataset. It is merged into seven land types according to the corresponding relationship in Table 1 and resampled to 1-km resolution. We assumed that physical conditions, such as topography, temperature, precipitation and soil, did not significantly change in two decades. Similarly, socioeconomic factors such as GDP and population did not significantly change in the spatial distribution pattern. Therefore, we employed the same spatial driving factor dataset to estimate the seven land types’ suitability probabilities when conducting historical land simulations. Then, we conducted historical land simulations by taking the observed area in 2015 as the land demands.

#### Accuracy of the suitability probability

**Fig. 5:**
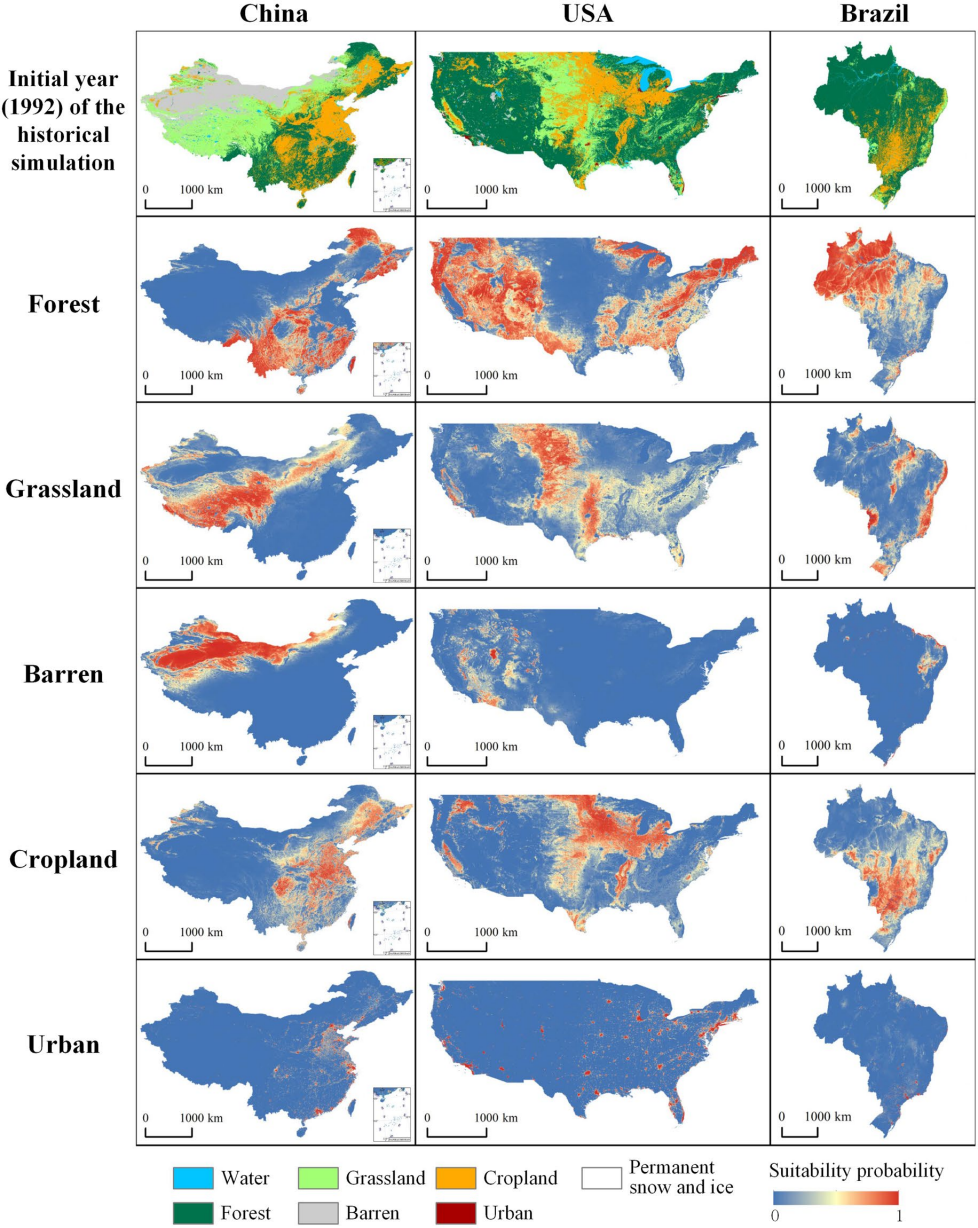
Comparison of suitability probability and observed land pattern for representative regions in the initial year of the historical simulation.

Suitability probability is an essential part of the FLUS model. As shown in Fig. 5, we selected three representative regions (China, USA and Brazil) containing comprehensive land types to exhibit the spatial distribution of suitability probabilities in the historical land simulation. The results show that the spatial distributions of the suitability probabilities achieve good results in each region. Moreover, the suitability probability distribution of each land type generally coincided with the observed land pattern. The suitability probability’s spatial pattern shows that the suitability probability is relatively high in places where a specific land type is concentrated. However, the converse is observed in places where specific land types are scattered.

To quantify the suitability probability’s accuracy, we employed the receiver operating characteristic (ROC) curve as a detection tool. The area under the curve (AUC) from the ROC curve was used to measure the suitability probability’s accuracy. AUC ranged between 0 and 1. The larger the value, the higher the accuracy. We uniformly sampled each land type according to 10% of the total number of grids in each region and evaluated the accuracies shown in Table 5. Figures S8–S12 present the spatial distribution of AUC for different land types and regions. The average AUC values of the five main land types were above 0.91. Among them, the average AUC of urban land was the highest, reaching 0.959. The AUCs of barren and forest reached 0.942 and 0.941, respectively, while those of cropland and grassland reached 0.926 and 0.914, respectively. Among the various land types in each region, the OAS-M region’s grassland (other middle and high-income Asian countries, including Singapore, Malaysia, Thailand and other Southeast Asian countries) afforded the lowest AUC value (0.821). Since less grassland is present in this region, this low AUC value has a limited impact on the land simulation’s accuracy. Generally, the AUC of the suitability probabilities reached the desired accuracy in each region and each land type.

**Table 5:** AUC of the suitability probability in each region and each land type.

Region	Forest	Grassland	Barren	Cropland	Urban
ANUZ	0.951	0.933	0.936	0.980	0.942
BRA	0.928	0.942	0.927	0.918	0.970
CAN	0.979	0.954	0.989	0.979	0.931
CAS	0.906	0.929	0.948	0.864	0.960
CHN	0.969	0.969	0.986	0.950	0.989
EEU	0.929	0.912	0.947	0.918	0.968
EEU-FSU	0.930	0.852	0.923	0.903	0.954
EFTA	0.926	0.932	0.973	0.944	0.971
EU12-H	0.881	0.892	0.941	0.889	0.964
EU12-M	0.927	0.884	0.939	0.907	0.972
EU15	0.943	0.919	0.957	0.933	0.984
IDN	0.949	0.973	0.919	0.889	0.928
IND	0.970	0.920	0.937	0.965	0.978
JPN	0.962	0.925	0.888	0.921	0.983
KOR	0.959	0.860	0.852	0.856	0.950
LAM-L	0.944	0.951	0.875	0.932	0.928
LAM-M	0.945	0.920	0.986	0.963	0.984
MEA-H	0.837	0.812	0.982	0.903	0.970
MEA-M	0.905	0.915	0.942	0.911	0.970
MEX	0.952	0.957	0.979	0.896	0.982
NAF	0.964	0.933	0.950	0.945	0.922
OAS-CPA	0.965	0.952	0.980	0.931	0.909
OAS-L	0.969	0.938	0.884	0.920	0.911
OAS-M	0.974	0.821	0.965	0.963	0.988
PAK	0.938	0.930	0.906	0.965	0.988
RUS	0.957	0.906	0.962	0.982	0.921
SAF	0.932	0.918	0.981	0.933	0.981
SSA-L	0.950	0.946	0.993	0.918	0.922
SSA-M	0.925	0.941	0.987	0.935	0.944
TUR	0.926	0.903	0.899	0.889	0.991
USA	0.950	0.882	0.976	0.938	0.973
Average	0.941	0.914	0.942	0.926	0.959

#### Accuracy of the land simulation

**Fig. 6:**
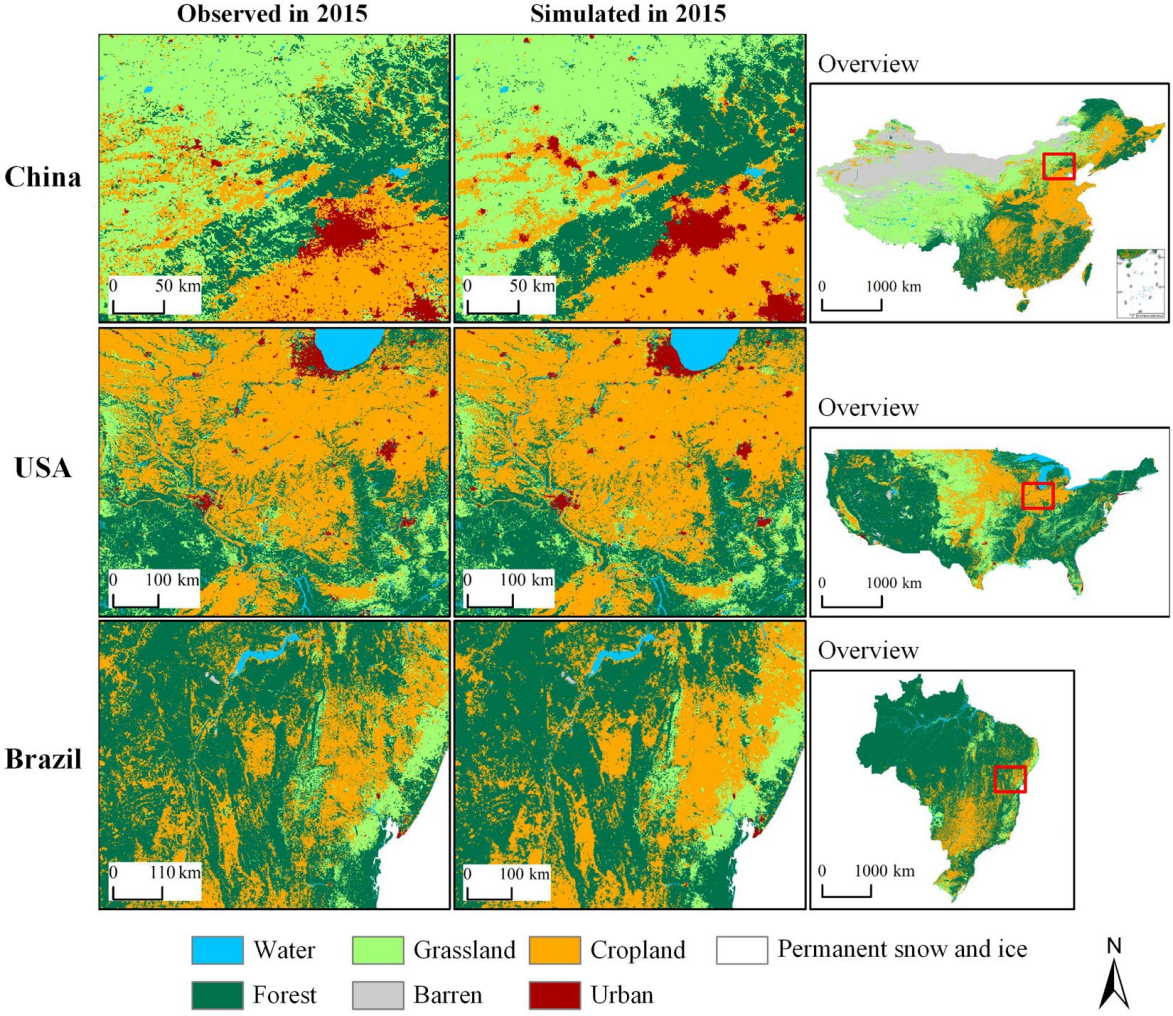
Comparison between observed and simulated land patterns for the representative regions in 2015.

Fig. 6 shows the comparison of the land pattern in 2015 obtained from the historical land simulation and the observed land data. The figure shows that the simulated land pattern is similar to the observed land pattern. The land simulations perform reasonably well. To further quantitatively evaluate the land simulation’s accuracy, the three commonly used accuracy indicators, the Kappa coefficient, overall accuracy (OA) and Figure of Merit (FoM), were used.

Table 6 lists each region’s Kappa coefficient, OA and FoM in the historical land simulation. The spatial distributions are shown in Figures S13–S15. From the overall results of the 31 regions, the Kappa coefficient is 0.864, OA is 0.929 and FoM is 0.102. Thus, the simulations afford high accuracy in terms of the Kappa coefficient and OA. However, the low FoM needs to be examined. According to related literature, an FoM value in the 0.1–0.3 range is an acceptable result^17,45^, because FoM stringently measures the simulation accuracy. It only evaluates the part of the land that changes. Moreover, the FoM value is proportional to the proportion of land observed to change in the simulated region. This proportional relationship can generally reach 1.5:1. That is, when the proportion of land observed to change is 10%, the FoM of a good simulation result can reach 0.15^46^. In our historical land simulations, the observed global land changed from 1992 to 2015 accounted for only 3.10%, but the average FoM value reached 0.102. This accuracy is better than the general proportional relationship level, indicating that our land simulation’s accuracy is acceptable.

**Table 6:** Kappa coefficient, OA and FOM of the historical land simulation in each region.

	Kappa	OA	FoM	Region	Kappa	OA	FoM
ANUZ	0.889	0.925	0.044	LAM-M	0.887	0.937	0.055
BRA	0.794	0.908	0.066	MEA-H	0.839	0.993	0.104
CAN	0.962	0.976	0.179	MEA-M	0.898	0.961	0.125
CAS	0.836	0.880	0.112	MEX	0.919	0.967	0.103
CHN	0.847	0.886	0.137	NAF	0.903	0.986	0.123
EEU	0.841	0.911	0.100	OAS-CPA	0.895	0.928	0.074
EEU-FSU	0.763	0.896	0.066	OAS-L	0.874	0.940	0.084
EFTA	0.914	0.935	0.070	OAS-M	0.885	0.940	0.109
EU12-H	0.856	0.919	0.057	PAK	0.835	0.881	0.133
EU12-M	0.856	0.918	0.056	RUS	0.891	0.943	0.096
EU15	0.870	0.913	0.075	SAF	0.858	0.922	0.063
IDN	0.810	0.925	0.080	SSA-L	0.911	0.940	0.083
IND	0.918	0.959	0.093	SSA-M	0.914	0.959	0.160
JPN	0.773	0.903	0.213	TUR	0.876	0.920	0.085
KOR	0.677	0.818	0.215	USA	0.937	0.961	0.102
LAM-L	0.843	0.945	0.085	Average	0.864	0.929	0.102

#### Comparison of land cover representation in 1-km and coarse resolutions

**Fig. 7:**
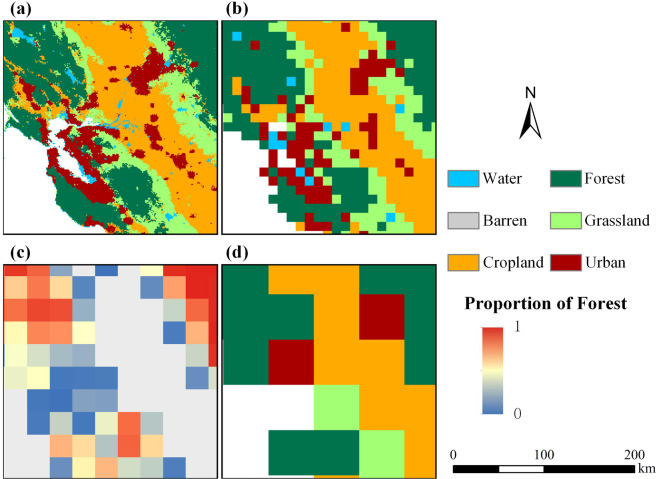
Differences in land spatial pattern representations using different resolutions (case of SSP2-4.5 in 2050 for the San Francisco metropolitan area, USA). (**a**) 1-km resolution; (**b**) 10-km resolution; (**c**) 25-km resolution (forest distribution shown in fractional form) and (**d**) 50-km resolution.

To test the superiority of our global LUCC product relative to the existing global LUCC products in terms of the spatial resolution, we selected a small-scale region to compare the effects of using different resolutions. We chose several resolutions that are commonly used in current global LUCC products, including 5′ (approximately 10 km on the equator) used by IMAGE 3.0^13^, LUSs^12^ and CLUMondo model^47^, 0.25° (~25 km on the equator) in the fractional form used by LUH2^4^ and 0.5° (~50 km on the equator) used by IMAGE 2.4^48^. Fig. 7 shows a comparison of our 1-km resolution product and these coarse resolutions. Furthermore, Fig. 7 shows the simulation results for 2050 for the middle road (SSP2-4.5) of the eight scenarios, highlighting the San Francisco metropolitan area in the USA. In addition to showing the representation in our 1-km product (Fig. 7a), representations in the 10-km resolution (Fig. 7b), 25-km resolution in fractional form (Fig. 7c) and 50-km resolution (Fig. 7d) are shown via resampling. Clearly, the use of land products with 10-km or coarser resolution merges many small patches of urban land (shown in dark red in Fig. 7 a,b,d) into other land types, causing a loss of urban spatial detail. Moreover, the 10-km resolution results are unable to depict the spatially intertwined pattern in the zones where different land types transition. The 25-km resolution in fractional form also loses considerable spatial detail when describing the forest. For the 50-km resolution, the spatial distribution of land cover in this metropolitan area can be represented using only 20 or so grids.

### Performance of the simulation of future land dynamics

We created a 1-km resolution global land dataset under SSP-RCP scenarios at 5-year intervals from 2015 to 2100, comprising seven land types, through future land simulations. To strengthen the land change visualisation, we counted and displayed the area of land change (km^2^) on each 10 × 10 km^2^ grid by 2100.

**Fig. 8:**
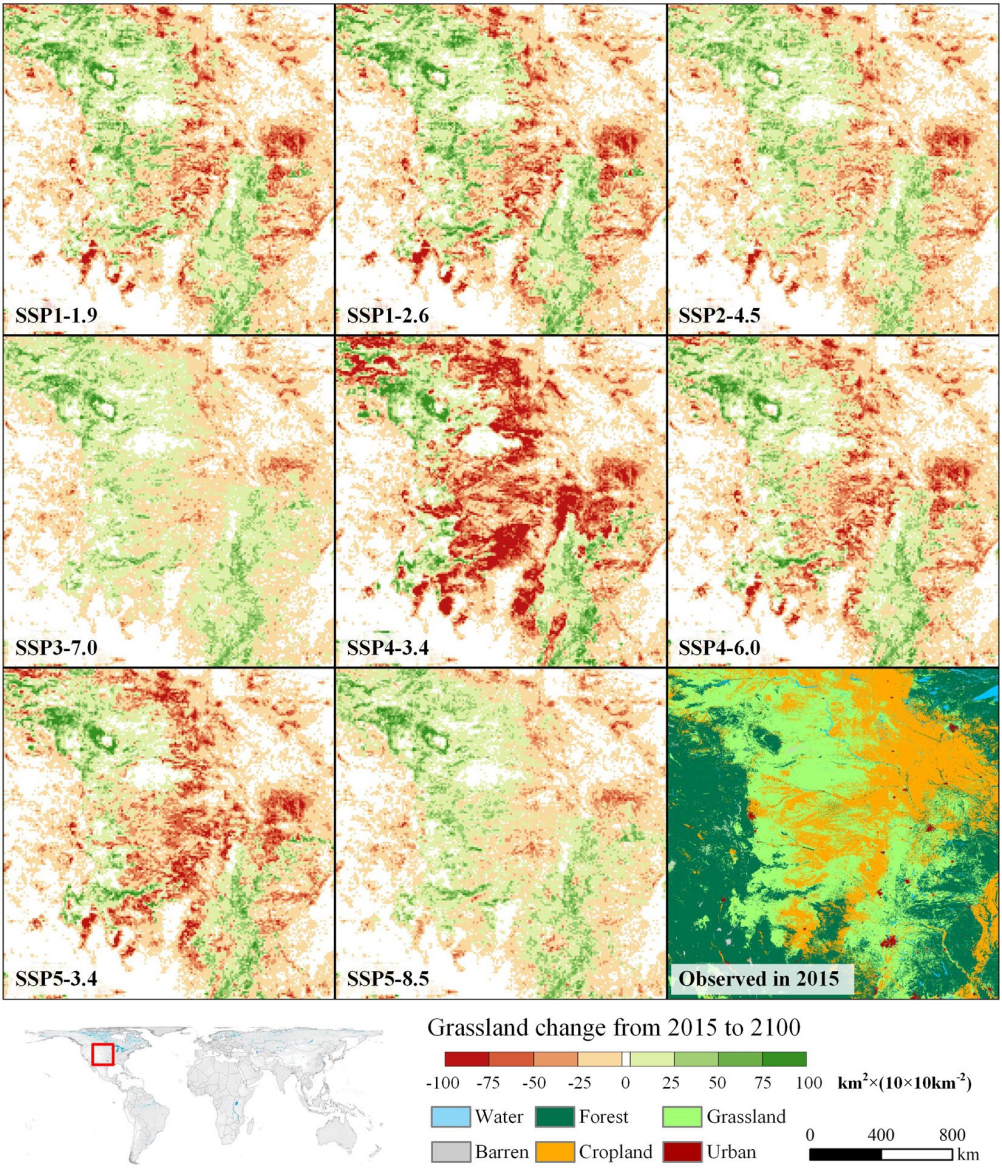
Grassland change in central USA under different SSP-RCP scenarios from 2015 to 2100. The colour from red to green represents the net change in grassland area in a 10 × 10 km^2^ grid from decreasing to increasing. The grassland in the white grid is frozen.

Taking grassland as an example, we selected the prairie in the central USA as a representative area to show its spatial changes between 2015 and 2100 (Fig. 8). The red grid in Fig. 8 represents grassland reduction, and the green grid represents grassland expansion. The figure clearly shows that different socioeconomic and climate policies affect grassland changes^4^.

Under the two coupled scenarios of SSP1, the green development road, animal product consumption reduction caused a decrease in pasture demand. Simultaneously, cropland increased with the use of biomass energy. Therefore, under the background of the total reduction of grassland in the USA, the grassland in the grassland–cropland junction area clearly decreased, but a relatively noticeable increase was observed inside the grassland area (SSP1-1.9 and SSP1-2.6 in Fig. 8). In the SSP3 scenario, although factors such as a high proportion of animals in the diet promote an increase in grassland (pasture), the USA has a slow-developing economy and the smallest population among the five SSP scenarios. The two opposing factors cause a slight rise in grassland demand. Therefore, the prairie of the central USA remains relatively stable under SSP3-7.0, and the areas of increase and decrease are balanced. Under the SSP5 scenarios, rapid population and economic growth generate a strong demand growth in food and feed. However, due to the rapid development of agricultural technology and the high intensification of the livestock production system, under SSP5-8.5, the USA’s grassland area remains stable, displaying a low intensity of grassland spatial changes (SSP5-8.5 in Fig. 8). However, under SSP5-3.4, to achieve the goal of net-zero CO_2_ emission, the scenario supposes that the second-generation bioenergy crops will be widely promoted after 2040^11^. Therefore, the grassland area in the USA will drastically decrease. This is manifested in the large-scale encroachment of grassland by cropland in the grassland–cropland junction area in central USA (SSP5-3.4 in Fig. 8). A similar situation also occurs in SSP4-3.4. Nevertheless, the difference is that cropland most severely encroaches on grassland due to its lack of an intensive production system (SSP4-3.4 in Fig. 8).

**Fig. 9:**
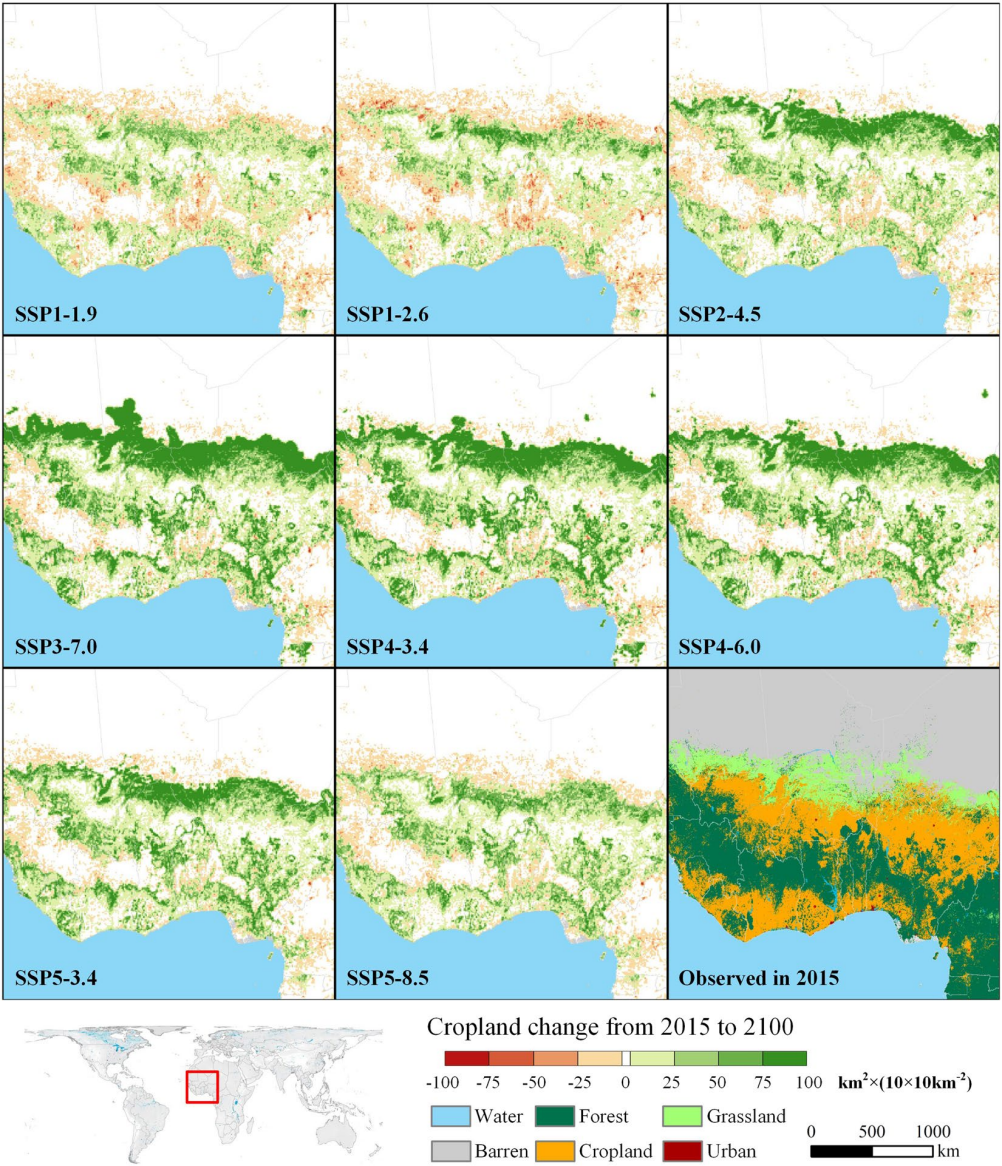
Cropland change in western Africa under different SSP-RCP scenarios from 2015 to 2100. The colour from red to green represents the net change in cropland area in a 10 × 10 km^2^ grid from decreasing to increasing. The cropland in the white grid is frozen.

We selected a cropland agglomeration area near the Gulf of Guinea in western Africa as a representative area to stimulate the spatial changes of cropland from 2015 to 2100 (Fig. 9). This area is located in the SSA-L region (i.e. a low-income country in sub-Saharan Africa), and it is the main cropland production area in SSA-L. As SSA-L is expected to have a substantial population growth ranging from 98% to 340% under all SSPs by 2100^49^, food demand is expected to inevitably increase. Therefore, the cropland areas of SSA-L increase in varying degrees under different SSPs. Notably, in SSP3-7.0, a regional competition and confrontation scenario, SSA-L exhibits the most dramatic population growth, slow technological development, and hindered international trade, causing the demand for cropland to skyrocket. Therefore, it is manifested as a substantial expansion of cropland to grassland and forest (SSP3-7.0 in Fig. 9). SSP4 is a polarisation scenario, making SSA-L, the low-income region, has many similarities in SSP4 and SSP3, such as substantial population growth. However, compared to SSP3, international trade will not be hindered in SSP4. Therefore, depending on the global food supply, SSA-L’s cropland demand is slightly lower in SSP4-6.0 than that in SSP3-7.0.

**Fig. 10:**
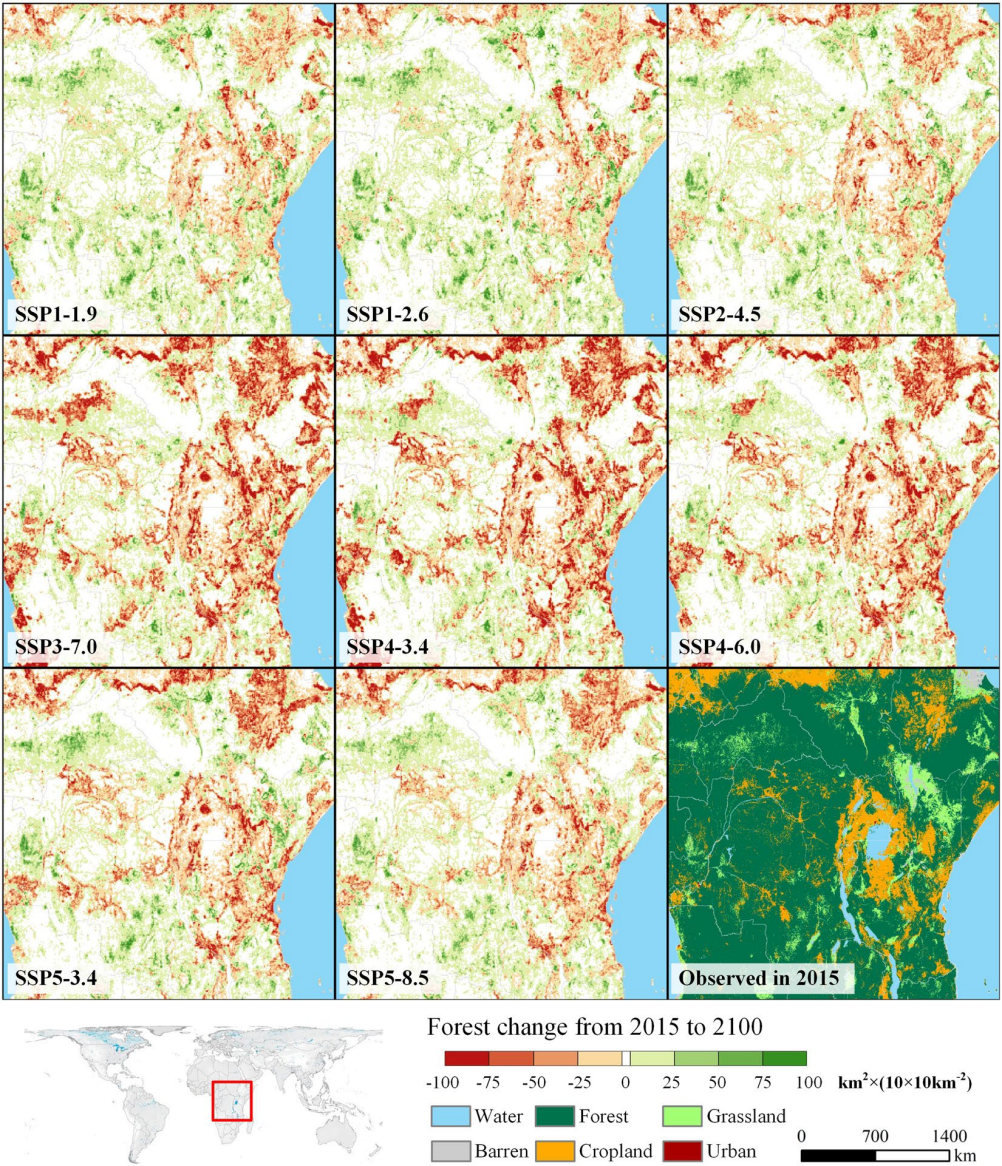
Forest change in central Africa under different SSP-RCP scenarios from 2015 to 2100. The colour from red to green represents the net change in forest area in a 10 × 10 km^2^ grid from decreasing to increasing. The forest in the white grid is frozen.

We also selected the forest agglomeration area in central Africa that is centred on the Congo Basin as a representative area to show the forest’s spatial changes from 2015 to 2100 under different SSPs (Fig. 10). The Congo Basin is one of the three major tropical rainforest areas in the world. The extensive tropical rain forest and surrounding forest in this area affect the regional as well as global ecosystems. The results show that in the green development road of SSP1 (SSP1-1.9 and SSP1-2.6), the forests in this area are extensively and well protected and the implementation of the biodiversity protection policy has restored them. In the three scenarios corresponding to SSP3 and SSP4, this area suffered the most severe and extensive forest degradation. This was mainly due to the rapid cropland expansion in SSA-L, a low-income region, in SSP3 and SSP4. Further, even the scattered cropland in the forest agglomeration area encroached a large amount of forest.

### Performance of the PFT-based land dataset

By subdividing the seven-land-type global land simulation products, we created a 1-km global land cover dataset under the SSP-RCP scenarios based on PFT classification with 20 land types from 2015 to 2100. Figure S16 displays an overview of the PFT-based land dataset.

**Fig. 11:**
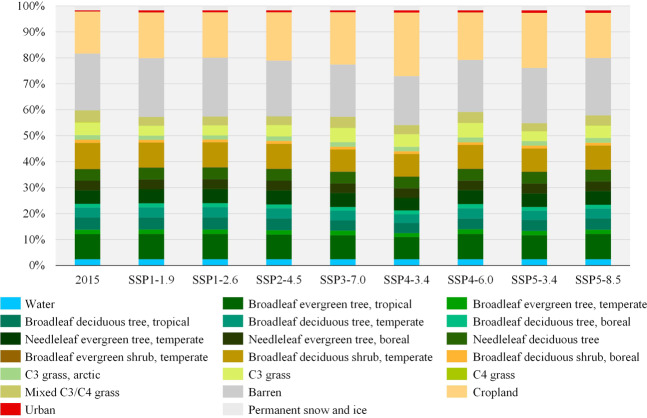
Comparison of the proportion of PFT land types under SSP-RCP scenarios in 2015 and 2100.

To clearly depict the land changes of each PFT under the SSP-RCP scenarios, the proportion of various land types in our global PFT dataset in 2015 and 2100 was compared (Fig. 11). The results show that among the land types, cropland fluctuates the most in different scenarios. The areas of various vegetations also correspondingly change. Additionally, the mixed C3/C4 grass exhibits a relatively obvious decrease in each scenario. This signifies that the encroachment on grassland owing to the cropland expansion mainly occurs near the warm temperate zone, i.e. areas with relatively good hydrothermal conditions. In SSP3-7.0, SSP4-3.4 and SSP5-3.4, where substantial cropland expansion occurs, the temperate broadleaf deciduous shrub, tropical broadleaf deciduous tree and temperate broadleaf deciduous tree relatively considerably decrease.

## Usage Notes

This study generated two 1-km future global LULC datasets from 2015 to 2100 with 5-year intervals under SSP-RCP scenarios, one comprising seven broad land types and the other comprising 20 PFT-based land types. The validation by performing historical land simulation revealed that the model affords excellent accuracy in all regions (on average, Kappa coefficient = 0.864, OA = 0.929 and FoM = 0.102). Moreover, our future datasets’ land changes appropriately reflect the storyline’s impact of SSP-RCP scenarios on the land cover. Therefore, we hope these two datasets, especially the PFT-based one, will better support environmental impact analysis and climate-related research under the latest climate scenarios.

Our datasets have the following advantages. First, due to the 1-km resolution, our datasets can map spatial details and reduce spatial uncertainty better than the existing global SSP-RCPs datasets, such as the 0.25° LUH2. Particularly, the spatial pattern of urban land is well preserved in our datasets. Second, our PFT-based dataset provides more plentiful land type information than the current fine-resolution future global land datasets, which usually contain only a few land types. Moreover, PFT-based land data are more valuable than broad land types data in the study of climate models. Therefore, our PFT-based dataset can be widely used in climate change research. Third, since our datasets adopt SSP-RCP scenarios and refer to the projected future land demand trajectories in LUH2, they are comparable with the official land dataset of CMIP6, which makes our datasets authoritative and universally applicable.

However, our datasets have several limitations. Users should evaluate whether these limitations affect them. First, the land cover classification accuracy from ESA-CCI data that was employed herein as the initial land data may yield potential errors in future land projections. Second, the spatial driving factors for future land simulation and the subdivision for the PFT-based dataset are not time varying, which is mainly limited by data availability. Moreover, spatial data for future global socioeconomic and soil drivers under SSP-RCPs are unavailable, and spatial data for future temperature and precipitation under SSP-RCPs suffer from coarse resolutions and insufficiently included scenarios. This limitation may cause local-scale deviations in depicting the land distributions from the scenario assumptions in our datasets, despite maintaining overall distribution reliability through suitability probabilities. We will resolve this limitation and update our datasets when the relevant spatial diver data based on SSP-RCPs become available. Third, like LUH2, water and permanent snow and ice in our datasets remain constant in the future. In reality, however, although they cover only a tiny fraction of the global continent, this may still have implications for researchers in specific fields, such as those concerned with snow and ice cover changes. Fourth, the spatial drivers used in our land simulation do not encompass all aspects, although various elements like socioeconomic, geomorphological, soil and climatic have been considered herein. For example, although precipitation, topography and soil quality, which can reflect water availability for crops to some extent, have been considered, they do not cover all the factors affecting cultivation, such as the distribution of agricultural infrastructure. Therefore, if a particular driver that a user is focusing on is not included in our spatial drivers, then our datasets may not be appropriate for them.

## Supplementary information

Supplementary information file

## Data Availability

The land simulation in this study was performed by the FLUS model software (GeoSOS-FLUS V2.4), which can be downloaded for free from http://www.geosimulation.cn/FLUS.html. Meanwhile, the tutorial on the operation of this software can be found in the user manual at this URL. The other spatial calculations and analyses in this study were performed by ArcGIS software as described in the Method section. The spatial data used for input are all publicly available online, with sources cited within the manuscript.
