# Supplementary material for: Global land projection based on plant functional types with a 1-km resolution under socio-climatic scenarios
Source: Sci Data. 2022 Mar 30;9:125. doi: 10.1038/s41597-022-01208-6 (PMC8967933; doi:10.1038/s41597-022-01208-6)
Supplement: Supplementary file 1 — Supplementary information file [file 41597_2022_1208_MOESM1_ESM.docx]

**Supplement of**

**Global land projection with a 1-km resolution based on the** **coupling scenarios of socioeconomic and climate change**

Guangzhao Chen^1,2,#^, Xia Li^3,#^, Xiaoping Liu^1,*^

1. Guangdong Provincial Key Laboratory of Urbanization and Geo-simulation, School of Geography and Planning, Sun Yat-sen University
2. Institute of Future Cities, The Chinese University of Hong Kong, Shatin NT, Hong Kong SAR
3. Key Lab of Geographic Information Science (Ministry of Education), School of Geographic Sciences, East China Normal University

# These authors contributed equally.

* Correspondence: Xiaoping Liu (liuxp3@mail.sysu.edu.cn)


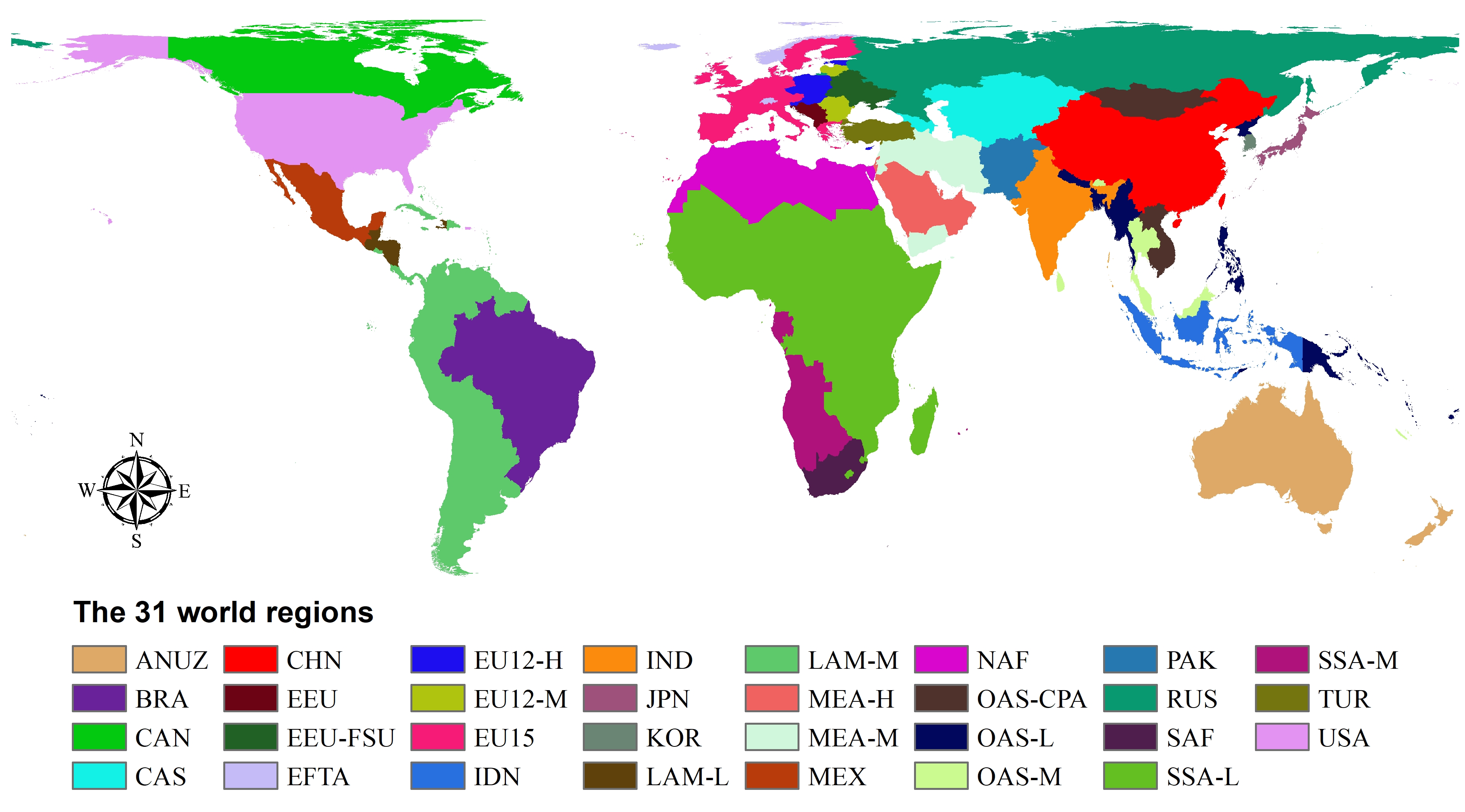


Figure S1 The 31 regions of the world


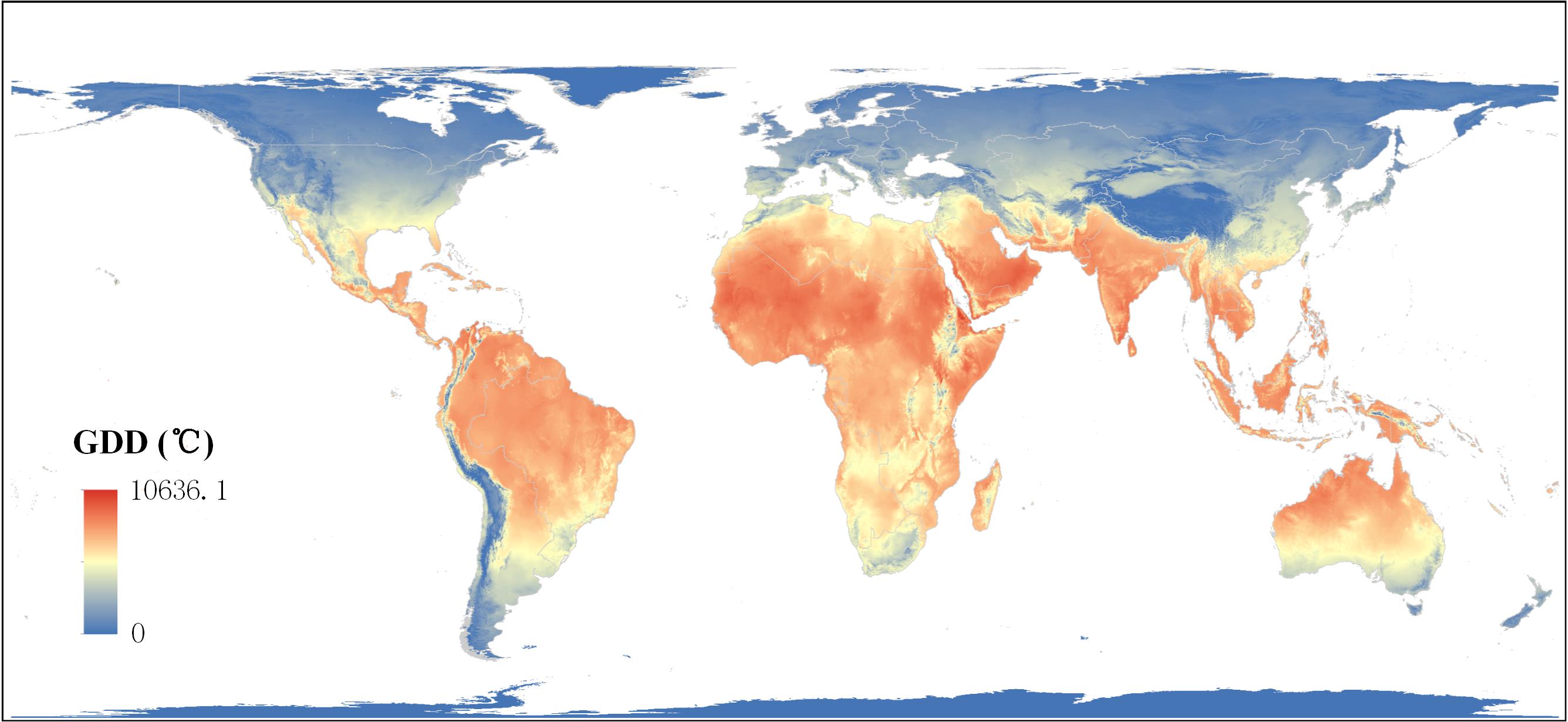


Figure S2 Historical average annual GDD from 1970 to 2000


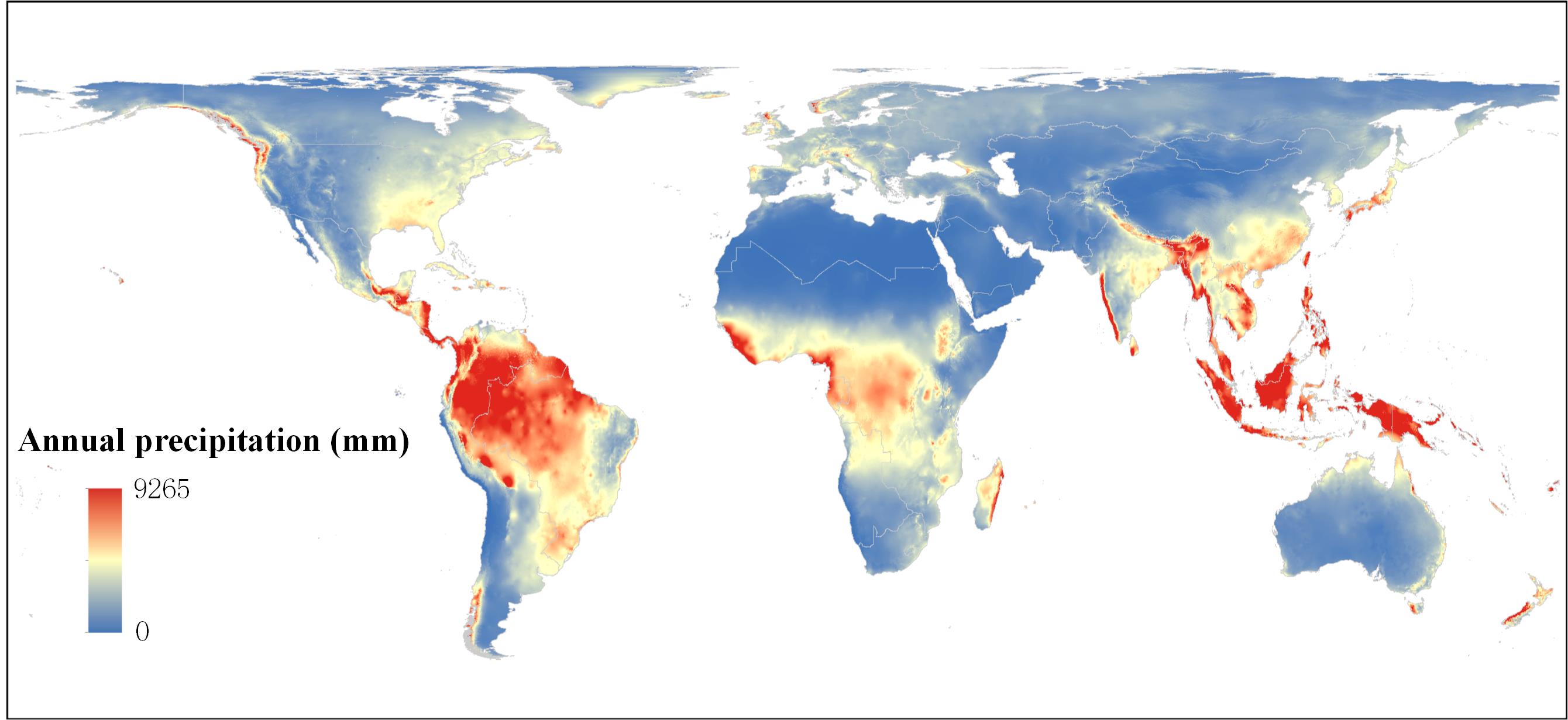


Figure S3 Historical average annual precipitation from 1970 to 2000


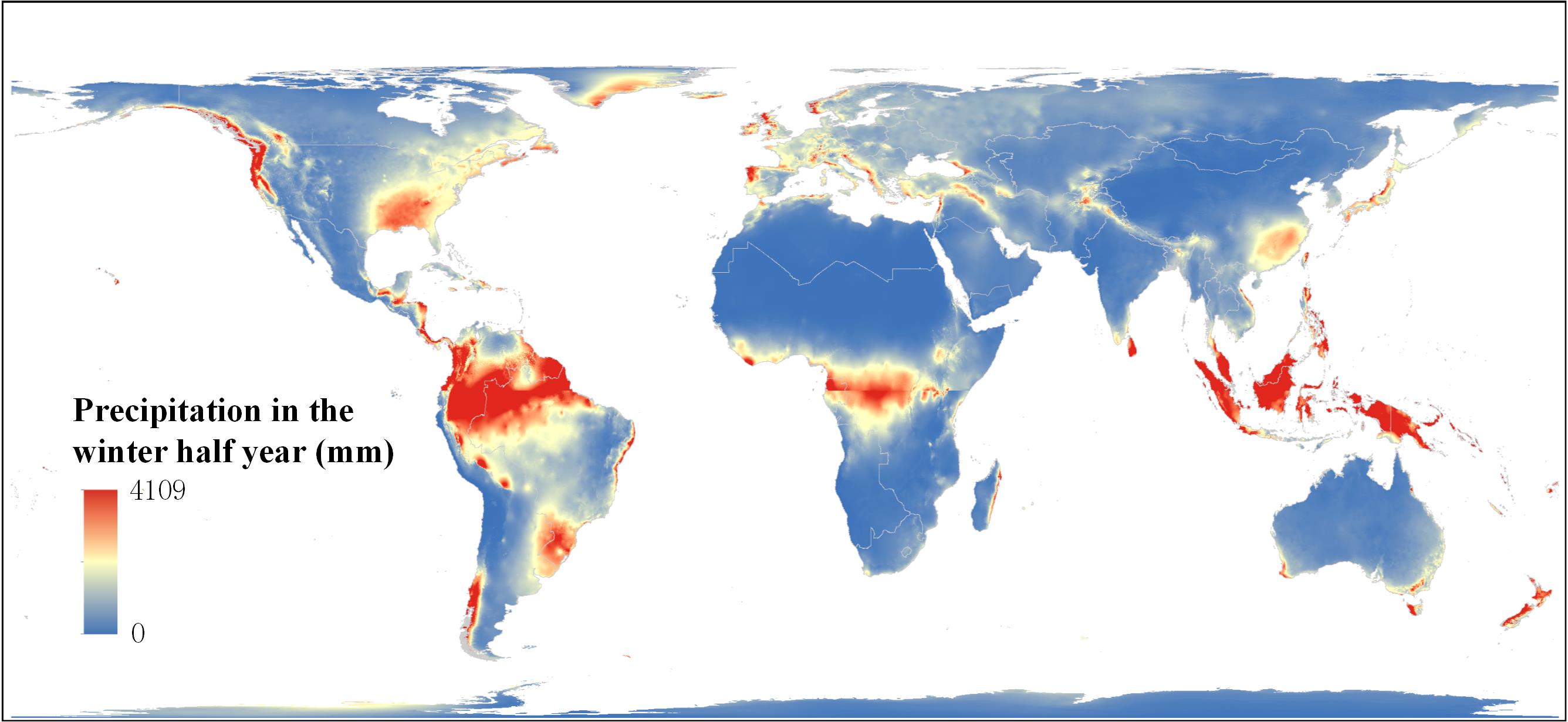


Figure S4 Historical average precipitation in the winter half year from 1970 to 2000


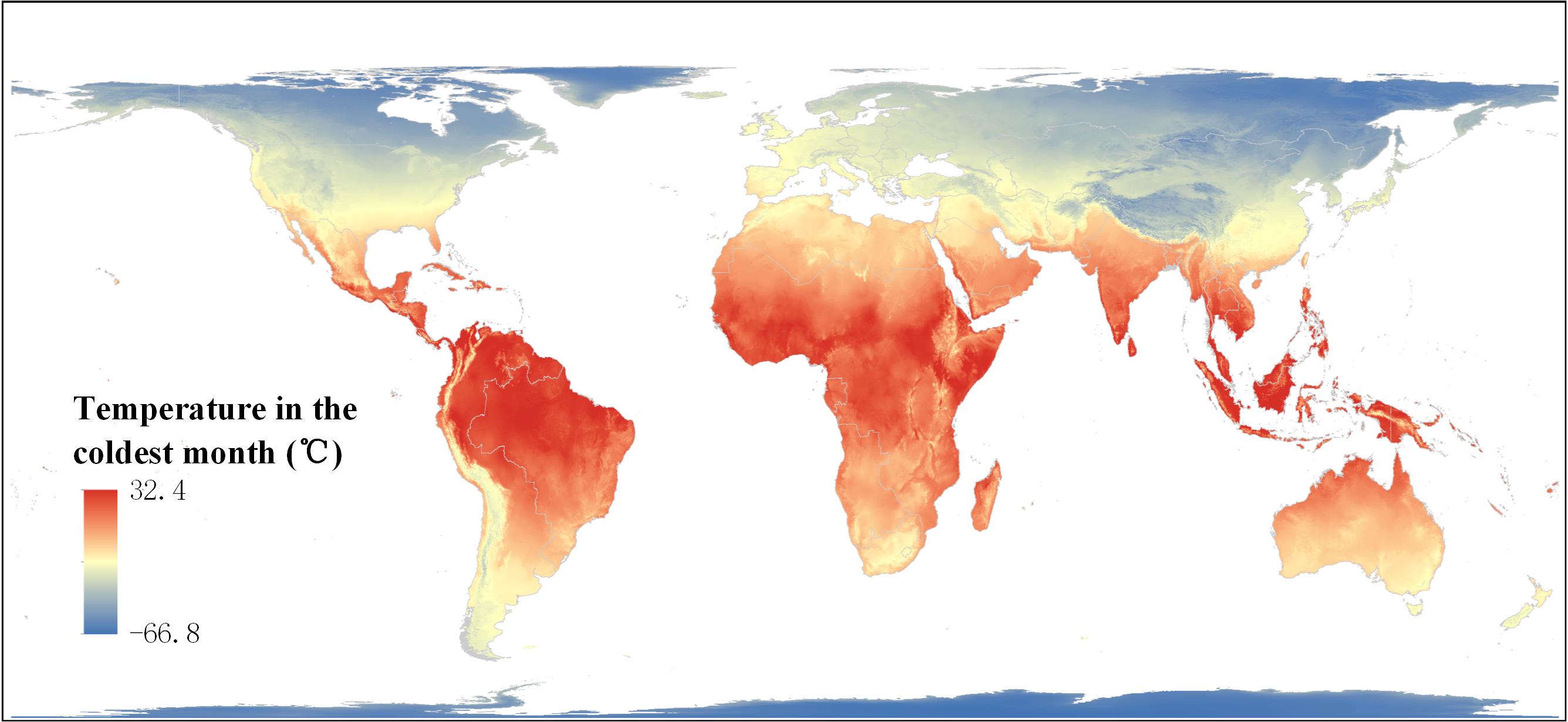


Figure S5 Historical average temperature in the coldest month from 1970 to 2000


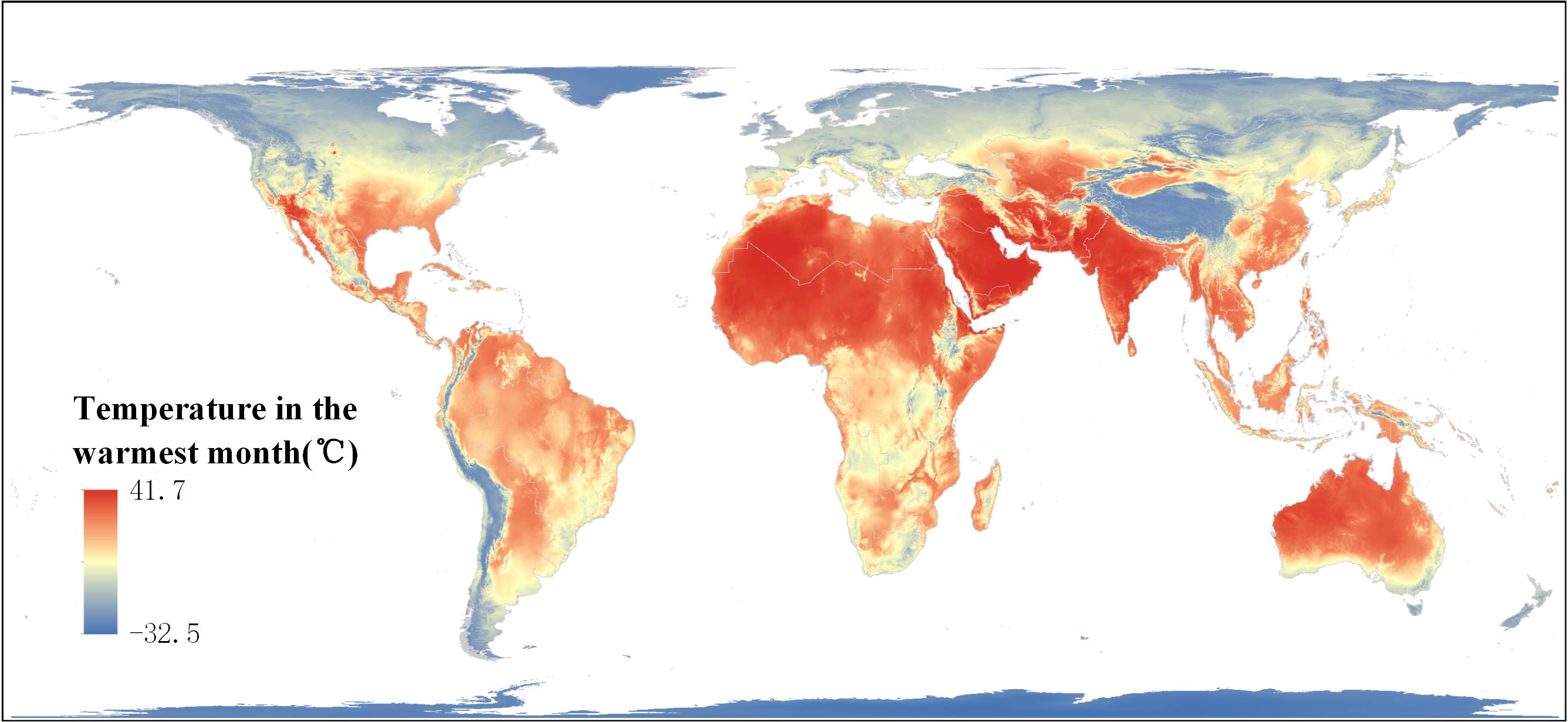


Figure S6 Historical average temperature in the warmest month from 1970 to 2000


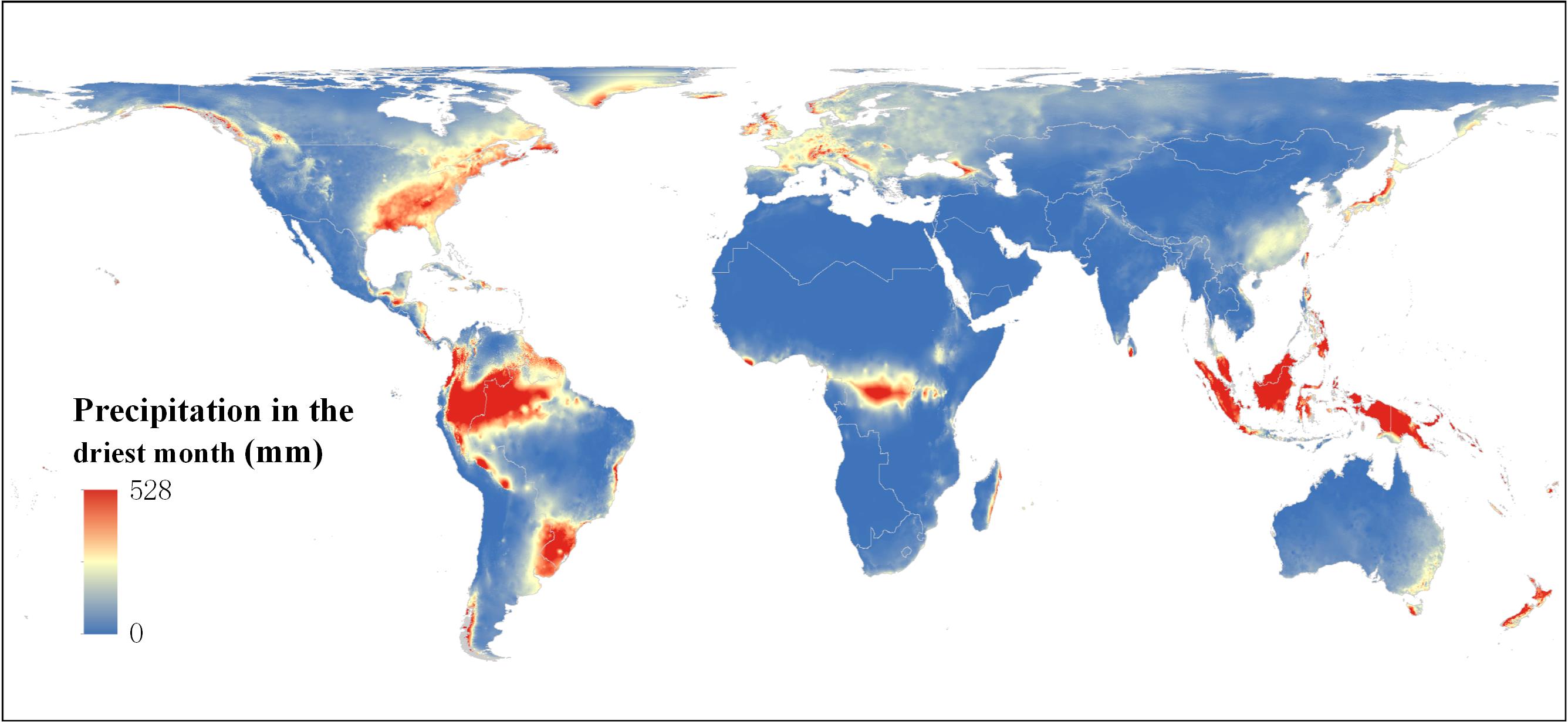


Figure S7 Historical average precipitation in the driest month from 1970 to 2000


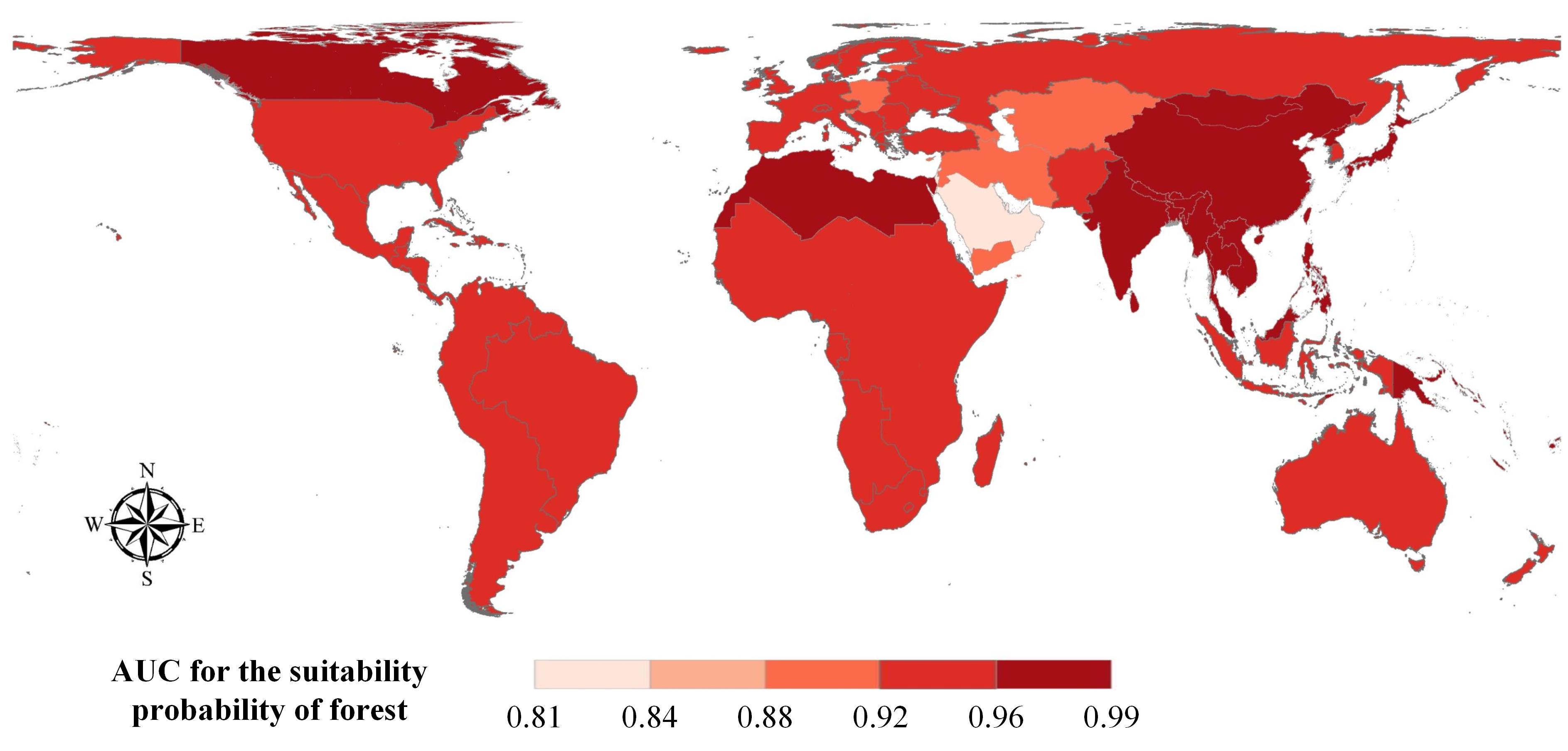


Figure S8 AUC for the suitability probability of forest


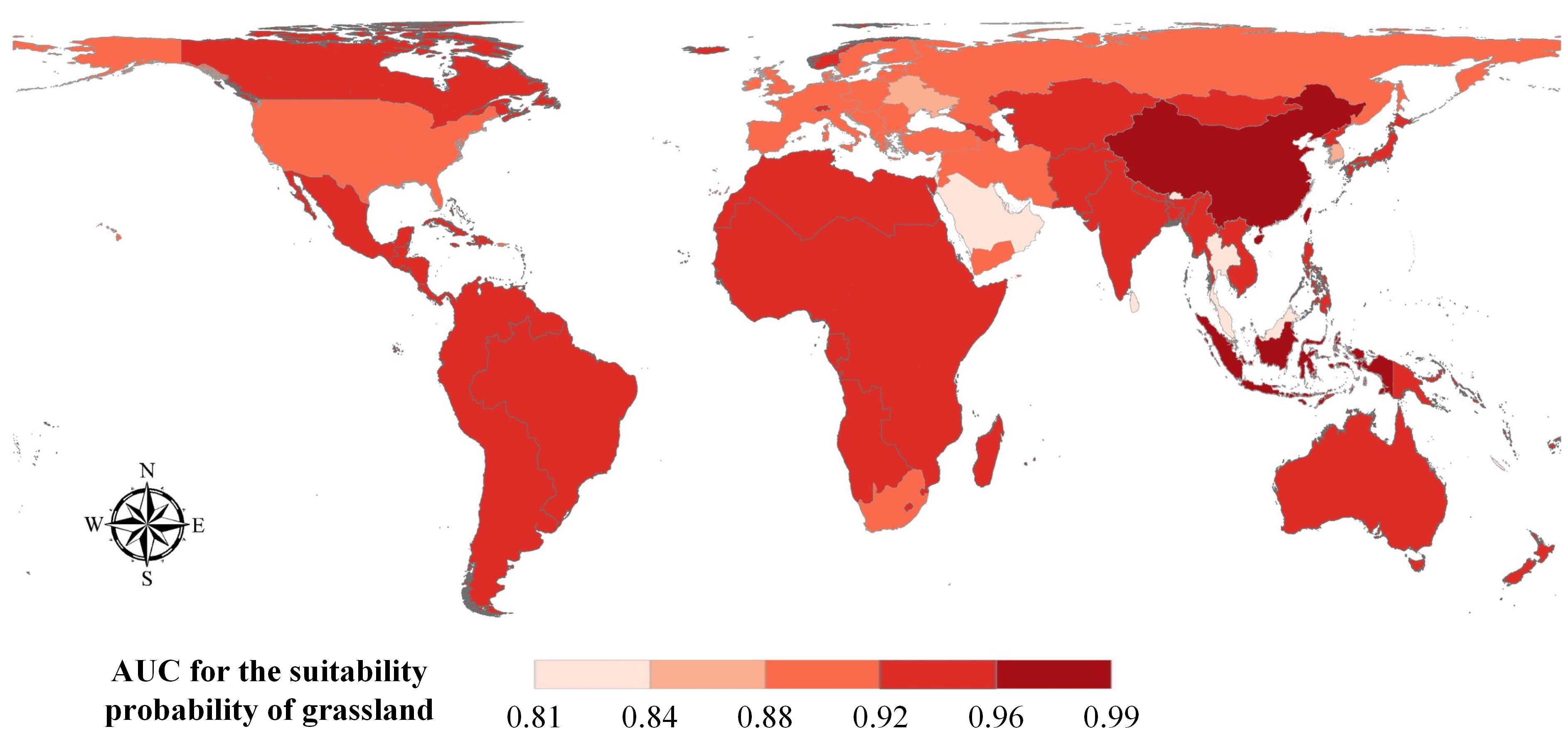


Figure S9 AUC for the suitability probability of grassland


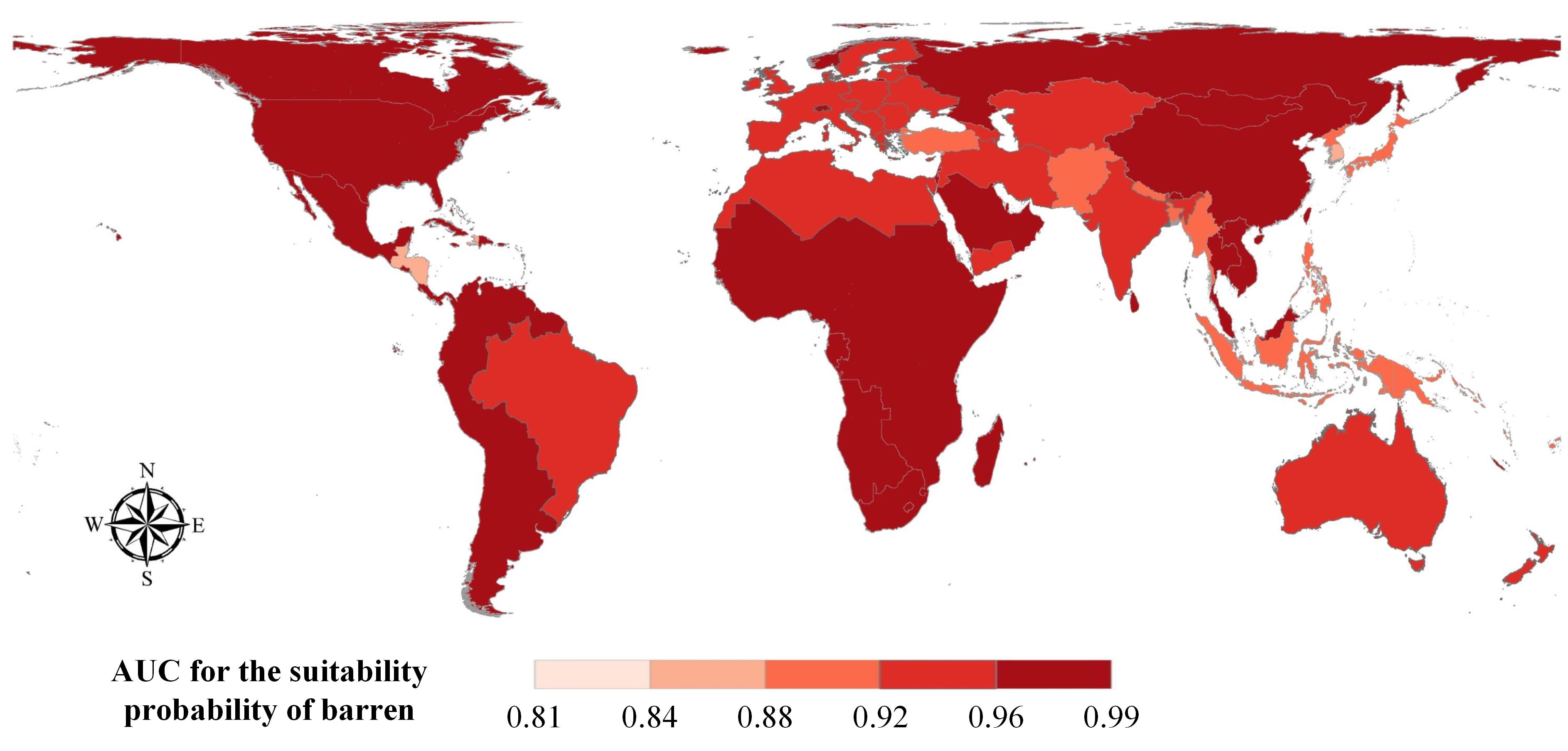


Figure S10 AUC for the suitability probability of barren


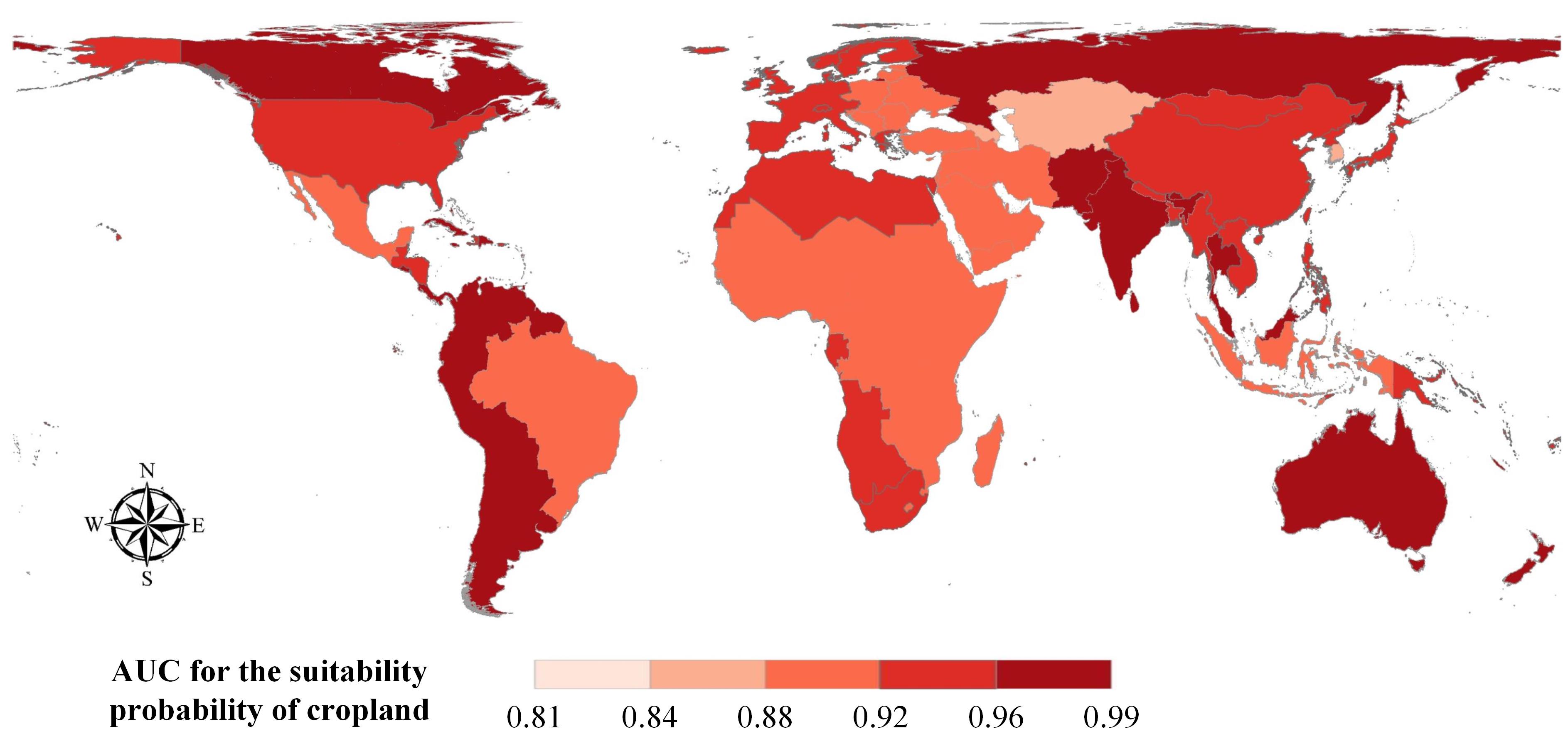


Figure S11 AUC for the suitability probability of cropland


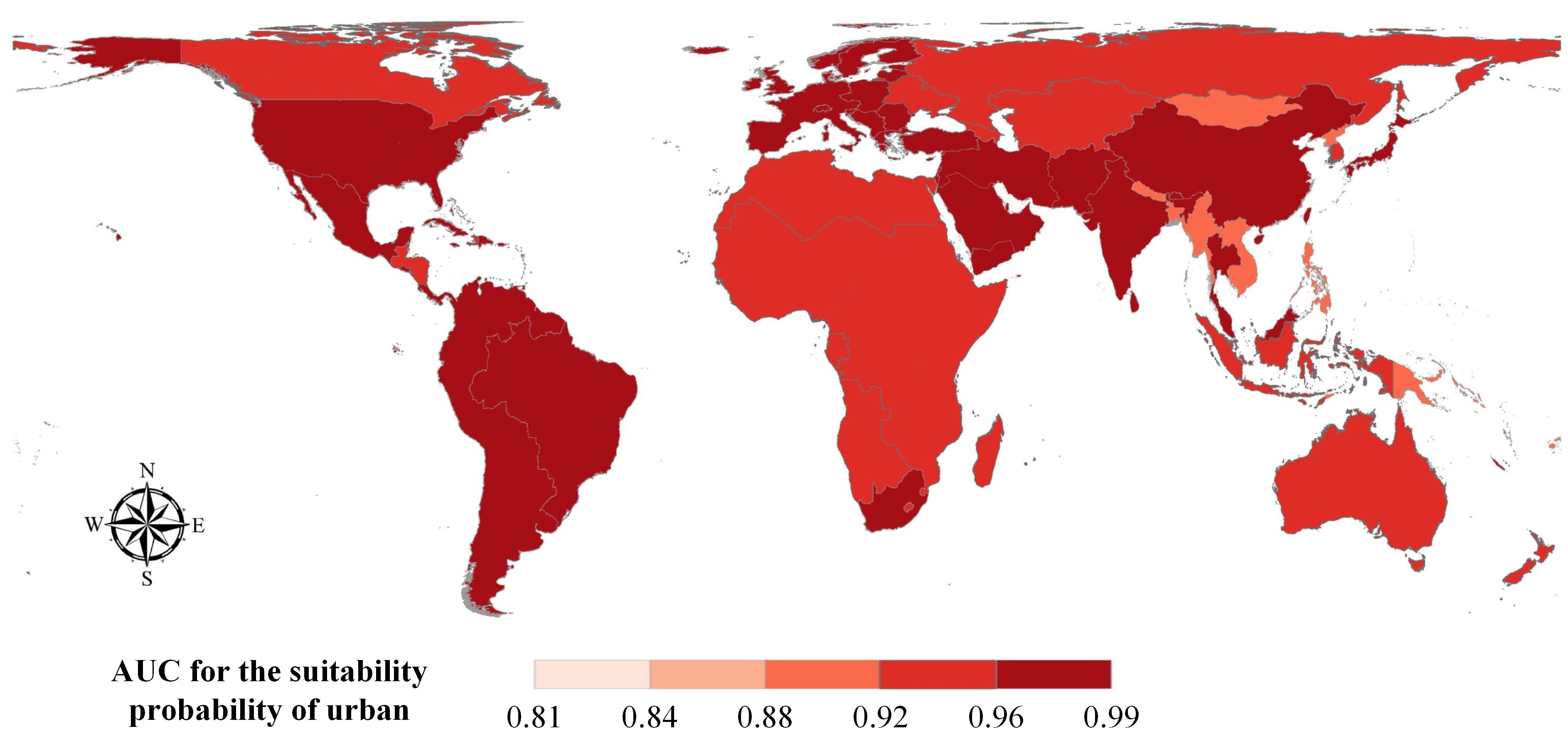


Figure S12 AUC for the suitability probability of urban


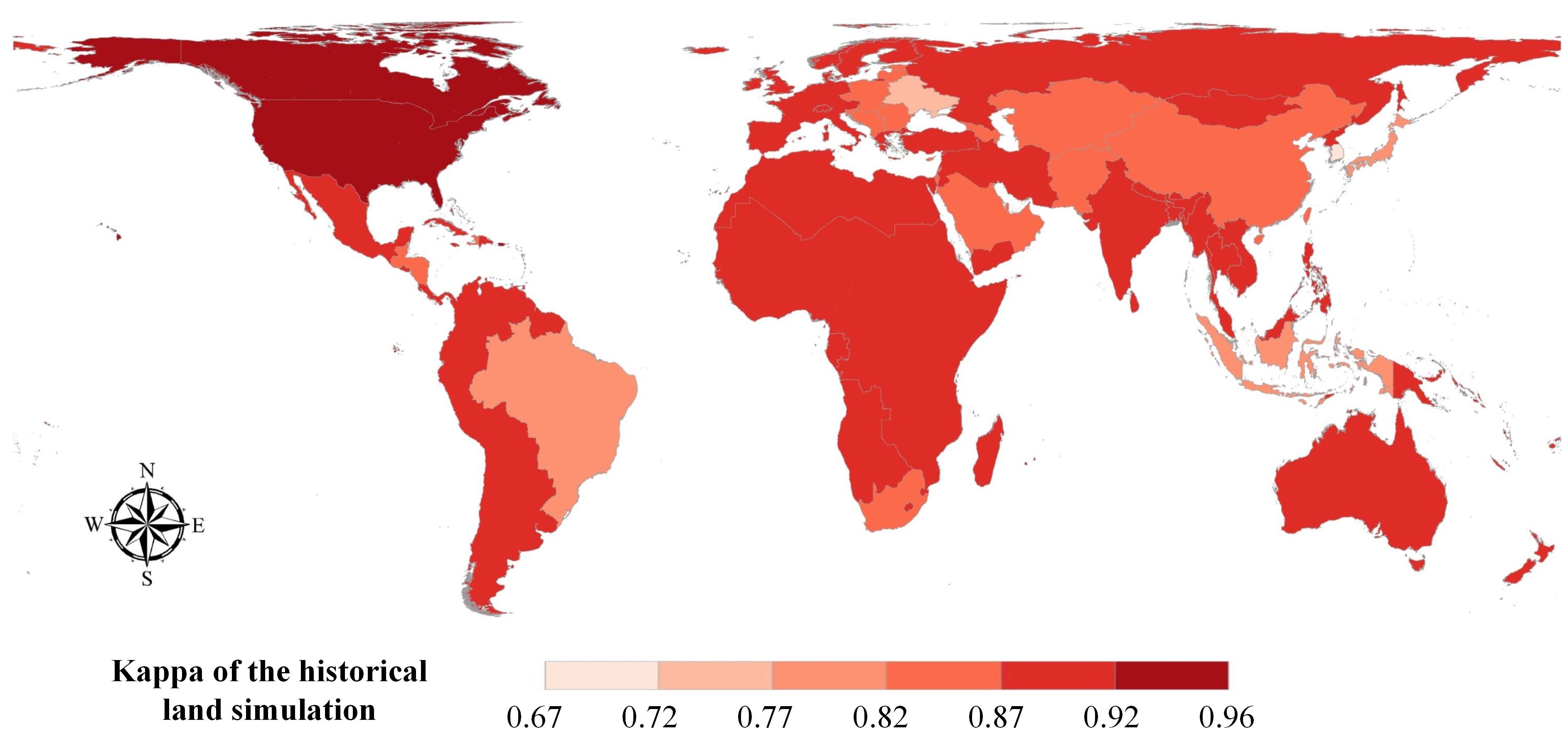


Figure S13 Kappa of the historical land simulation from 1992 to 2015


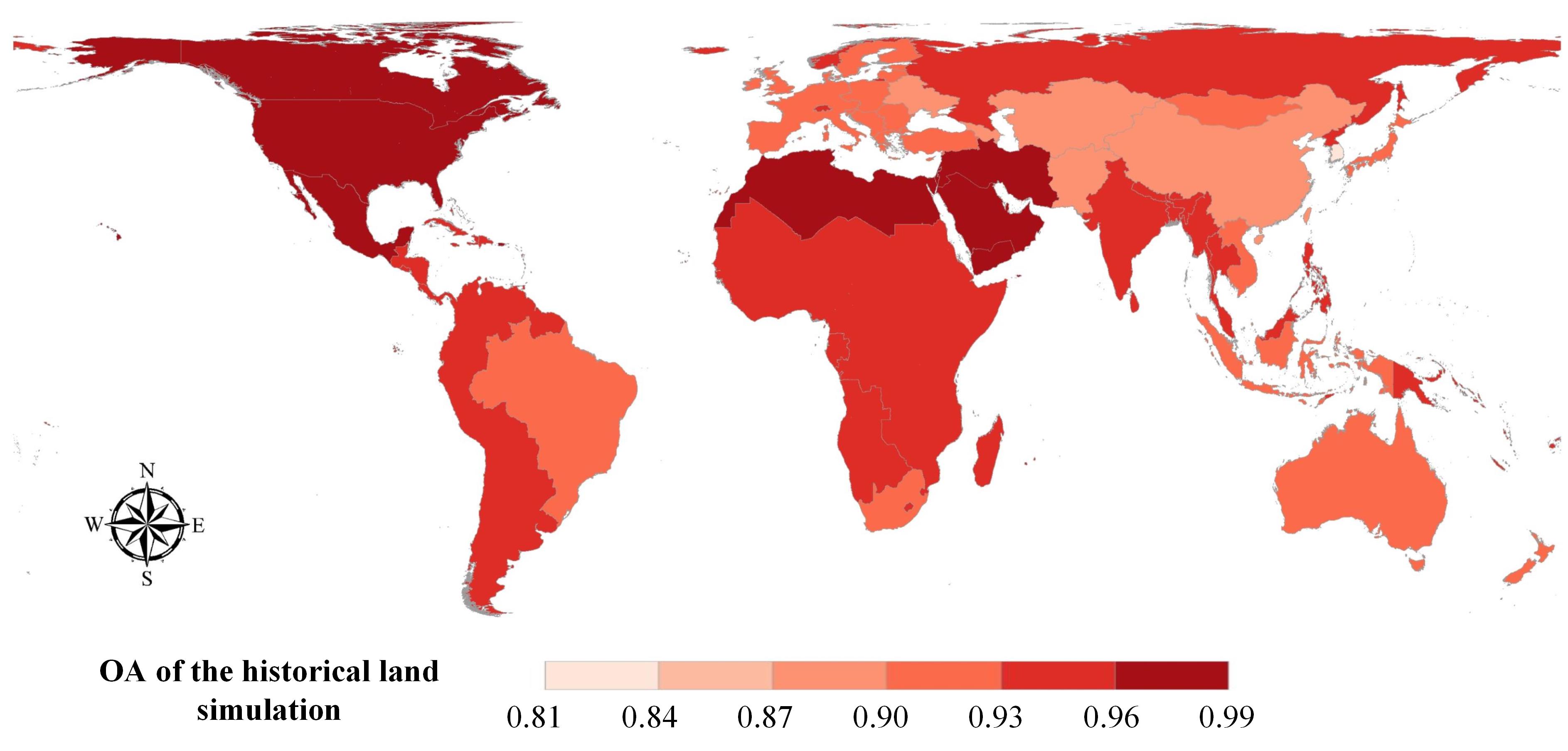


Figure S14 OA of the historical land simulation from 1992 to 2015


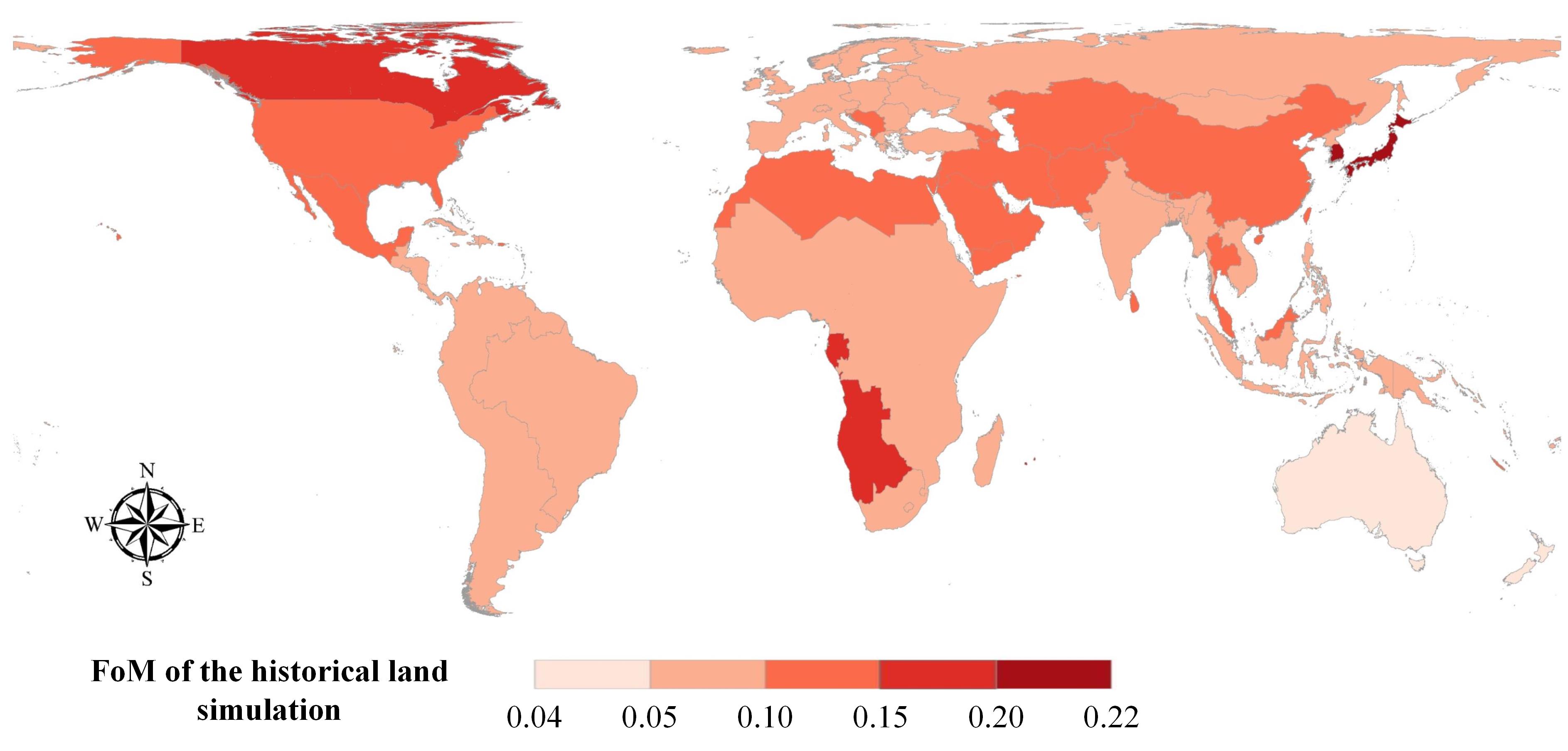


Figure S15 FoM of the historical land simulation from 1992 to 2015


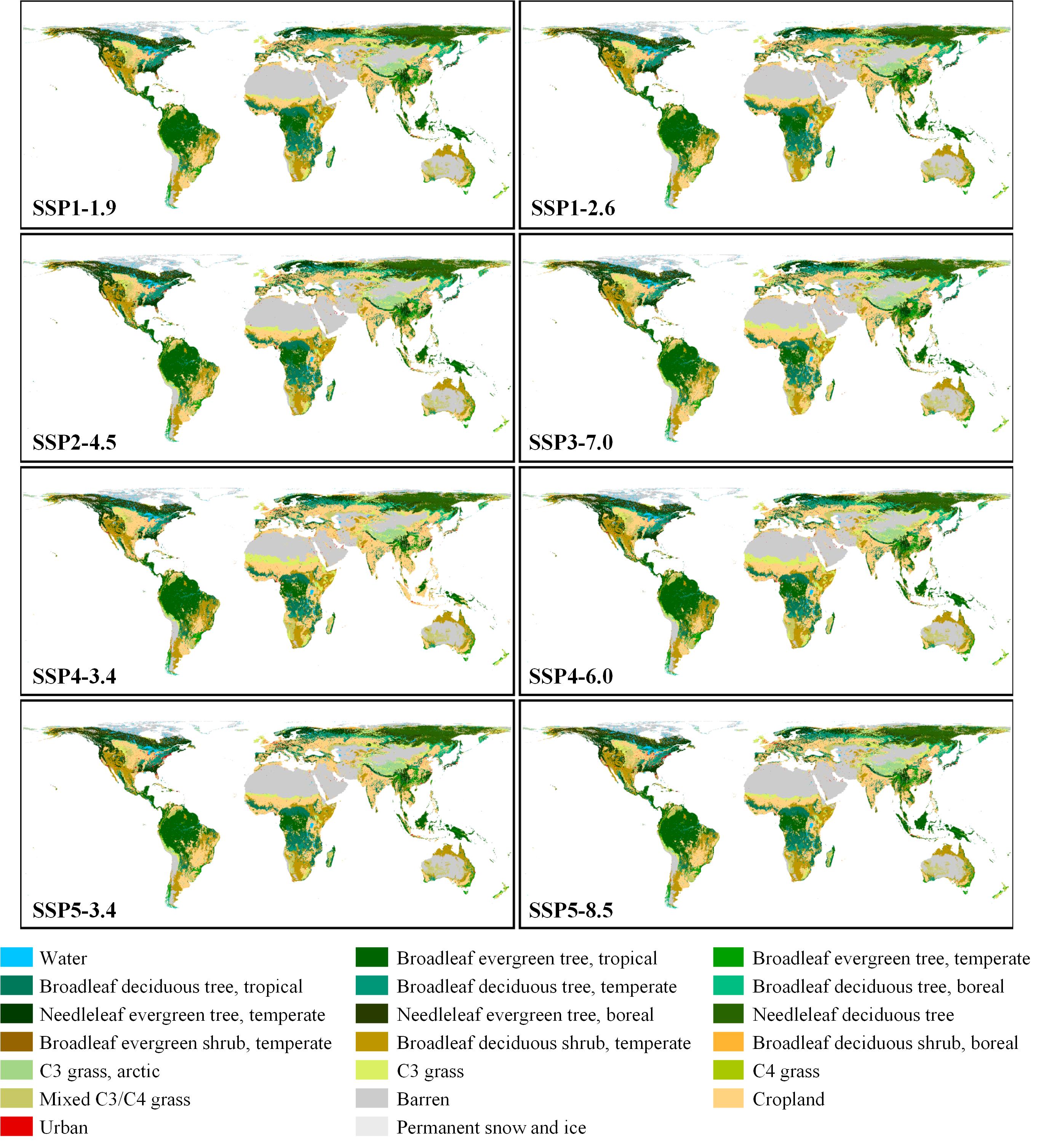


Figure S16 Performance of the PFT-based land dataset in 2100 under SSP-RCP scenarios
